# Developmental system drift in the patterning of the arthropod tarsus

**DOI:** 10.1098/rspb.2025.2557

**Published:** 2026-02-11

**Authors:** Benjamin Klementz, Sophie Neu, Ethan Laumer, Isaac Hinne, Emily Setton, Neeharika Verma, Max Hämmerle, John Rallis, Austen Barnett, Georg Brenneis, Monika Gulia-Nuss, Anastasios Pavlopoulos, Prashant Sharma

**Affiliations:** 1 Department of Integrative Biology, University of Wisconsin-Madison, Madison, WI 53706, USA; 2 Department of Biochemistry and Molecular Biology, University of Nevada Reno, Reno, NV 89557, USA; 3 University of Florida Whitney Laboratory for Marine Bioscience, St. Augustine, FL 32080, USA; 4 Marine Biological Laboratory, Woods Hole, MA 02543, USA; 5 Unit Integrative Zoologie, Department Evolutionsbiologie, Universität Wien, Vienna, Vienna 1030, Austria; 6 Foundation for Research and Technology Hellas, Institute of Molecular Biology and Biotechnology, Heraklion, Crete 70013, Greece; 7 Department of Biology, University of Crete, Heraklion, Crete 71500, Greece; 8 Department of Biology, Desales University, Center Valley, PA 18034, USA

**Keywords:** Chelicerata, Arachnida, appendage patterning, gene regulatory network, tarsomeres, Evolution, developmental biology

## Abstract

The current understanding of proximodistal axis patterning in arthropod legs is grounded in insect models. The paradigm for appendage evolution in this phylum is that the gene regulatory network responsible for leg subdivision and patterning is broadly conserved. Recent surveys of these genes have suggested that chelicerate exemplars exhibit divergent appendage patterning dynamics, though functional data remain limited. One salient mismatch in expression occurs in homologues of the homeobox gene *clawless*. In insects, *clawless* is expressed in the distalmost leg territory, specifying the claw-bearing pretarsus. In the harvestman *Phalangium opilio*, *clawless* occupies a broad tarsal domain early in development, localizing later to the metatarsus-tarsus boundary, suggestive of a tarsal patterning function. Here, we tested the function of harvestman *clawless* using RNA interference. Unlike insects, we show that *clawless* knockdown results in disrupted tarsal growth and patterning of its proximal segmental boundary, with no effect on the claw. Truncation of the tarsus is associated with defective tarsomere formation. We additionally surveyed the expression of *clawless* homologues in exemplars of chelicerate diversity, which suggests that the tarsal-patterning function for *clawless* was likely present in the most recent common ancestor of Chelicerata. These results join a suite of other comparative works suggesting that panarthropod appendage patterning exhibits numerous cases of developmental system drift.

## 1. Introduction

The hyperdiversity of extant arthropods is partly attributed to the modularity and adaptability of their segmented appendages. Almost every segment of the arthropod walking leg has been modified and outfitted with evolutionary novelties across the span of arthropod diversity. Further modifications of the walking leg developmental programme are understood to underlie the diversification of appendage types, such as insect mandibles, crustacean pleopods and sexually dimorphic gonopods in millipedes [1,2].

Much of what is known about appendage patterning in arthropods stems from seminal works in insect models such as the fruit fly *Drosophila melanogaster* and the red flour beetle *Tribolium castaneum*, which share the five-segmented condition archetypal of insects [3,4]. Work on satellite model systems has elucidated evolutionary dynamics of leg patterning, such as the role of different signalling pathways in leg segment morphogenesis [5–7]. Such datasets have broadly supported the interpretation that leg patterning is conserved across the phylum, with respect to: (i) the identity and spatial arrangement of transcription factor expression domains that establish the proximo-distal axis [8,9], (ii) the involvement of Notch-Delta signalling in establishing segment boundaries [10], and to a lesser extent, (iii) the identity of downstream genes that further subdivide the developing leg [11–15].

Drawing upon this pattern of conservation, a recent proposal has sought to align leg segments across the phylum through the lens of gene expression and functional data, with the goal of reconstructing the ancestral arthropod leg [13]. Our recent work, however, has highlighted the variability of leg patterning and gene expression dynamics across Chelicerata, the subdivision of arthropods that includes sea spiders and arachnids. Chelicerates are particularly notable for the anatomical variability of their appendage segment complement across extant orders, resulting in historical discord over their leg segment nomenclature. We were able to show that an intermediate leg segment, the patella, is established via a new expression domain of the homeobox gene *extradenticle* (*exd*) in a taxon-specific manner, with disruption of *exd* expression resulting in loss of the distal patellar boundary in a harvestman (*Phalangium opilio*; [16]). As an ancillary investigation, we also showed that many transcription factors known to pattern specific territories of the *D. melanogaster* leg exhibited markedly different expression domains in the developing legs of the harvestman and the sea spider *Pycnogonum litorale* [17].

**Figure 1.**
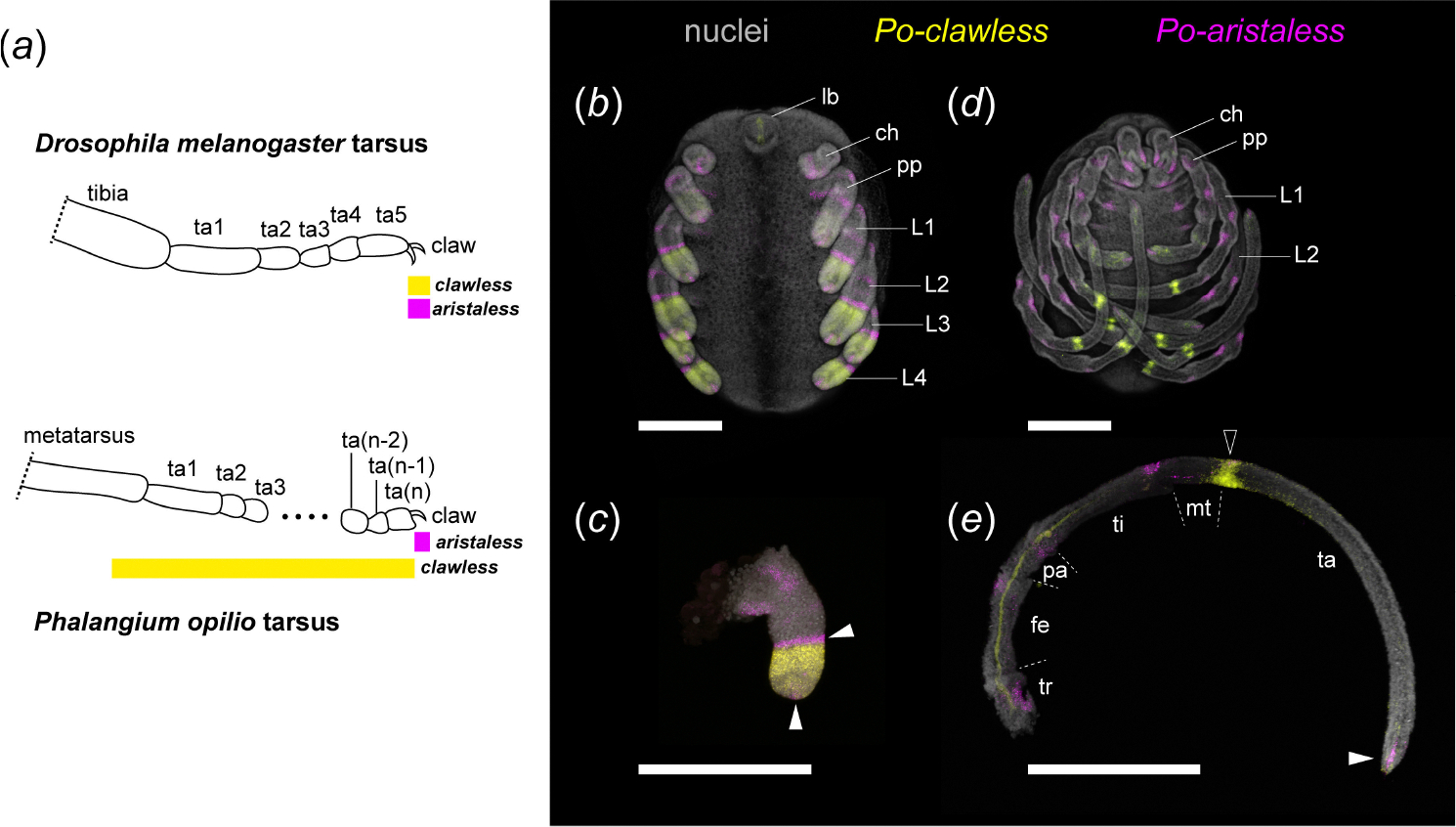
Divergent dynamics of distal limb patterning gene expression between arthropod taxa. (*a*) Schematic representations of *cll* and *al* expression domains in the distalmost podomeres of the *D. melanogaster* and *P. opilio* walking leg. (*b–e*) Multiplexed expression of *Po-cll* (yellow) and *Po-al* (magenta) with nuclear counterstaining (grey) in *P. opilio* embryos. (*b*) Stage 10 embryo. (*c*) Leg II of stage 10 embryo. Note the proximal expansion of *Po-cll* beyond *al*-positive cells in the distal terminus. (*d*) Stage 13 embryo. (*e*) Leg II of stage 13 embryo. Note the late localization of *Po-cll* to the metatarsal-tarsal boundary (black arrowhead) and depletion of tarsal expression. White arrowheads: *al*-positive expression domains. The proximal domain adjacent to *Po-cll* disappears by stage 13. Abbreviations: ch, chelicera; fe, femur; L, leg; lb, labrum; mt, metatarsus; pa, patella; pp, pedipalp; ta, tarsus; ti, tibia; tr, trochanter. Scale bars: 200 μm.

The most curious of these divergent expression domains pertains to the transcription factors *clawless* (*cll*) and *aristaless* (*al*) (figure 1a). In *D. melanogaster*, these two genes are required for the patterning of the claw-bearing pretarsus of the legs and of the arista of the antenna, and are co-expressed in the *Distal-less-*positive region of the imaginal disc that corresponds to the distal tip. The pretarsus is initially specified by activation of the homeobox transcription factors *BarH1* and *BarH2* (collectively, *Bar*), *al, cll* and *Lim1* as a product of distal-to-proximal epidermal growth factor receptor (EGFR) signalling gradients [15,18,19]. Al and Cll proteins bind cooperatively, likely to sites identified in the putative *Bar* enhancer, to repress *Bar* in the pretarsus, initially specifying the boundary between distal tarsus and proximal pretarsus. Likewise, *Bar* represses *Lim1* in the distal tarsus, itself necessary for further activation of *al* and *cll*, thus forming a regulatory feedback loop that leads to complete separation of tarsus and pretarsus [15,18,20–22]. Loss-of-function mutants for either gene exhibit a comparable phenotype, with losses of pretarsi and the distal part of the last tarsomere (in the legs) and of the aristae (in the antennae).

In marked contrast, in the harvestman, we found that *al* retains a limited expression domain in the tips of the walking legs (comparable to insects), whereas *cll* was broadly expressed throughout the distal limb bud in early stages of leg elongation, but was absent from the distalmost tip of the territory marked by *al*-positive cells (figure 1b,c) [17]. Later in development, when podomere boundaries are visible, *cll* spans the distal metatarsus and proximal tarsus, with the highest intensity of expression at the segmental boundary (figure 1d,e), consistent with other surveys of *P. opilio cll* expression [23,24]. Similar expression domains were likewise observed in the appendages of the sea spider *P. litorale*, supporting the inference that the dynamics of these genes are not restricted to harvestmen [17].

The evolutionary divergence of distal patterning in insect and chelicerate legs is unexpected because the homology of the distalmost territory of the walking leg has never been questioned, nor have functional data (where available) ever suggested deviation of tarsal patterning across Arthropoda. The distal region of the arthropod appendage, the tarsal claw, seems to be retained across all arthropod taxa, despite variations in nomenclature. In terrestrial lineages, single or paired tarsal claws (e.g. unguis) are borne on the distal terminus, the pretarsus (‘dactylus’ of crustaceans; ‘main claw’ or ‘dactylus’ of sea spiders), differentiated from true podomeres by the lack of intrinsic musculature (note: the pretarsal claws are commonly referred to as the tarsal claws in taxonomic literature of many arachnid groups; for the purpose of simplicity, we use the term tarsal claw here). However, in arachnids, the pretarsus is reduced to a ring-like sclerite operated by a single pair of antagonistic depressor and levator muscles [25]. The pretarsus can bear morphological modifications to improve traction reinforcement, such as the pulvillus or arolium, which facilitate adherence to smooth substrates. In spiders, additional claws serve the function of silk-handling hooks.

Here, we assessed the function of *cll* in the harvestman *P. opilio* using a gene silencing approach. Consistent with its expression pattern, we show that depletion of *cll* yields a fusion of metatarsus and tarsus, with truncation of the latter and loss of tarsomeres (articles of the tarsal segment), but has no effect on the tarsal claw. To assess the evolutionary dynamics of this gene, we surveyed *cll* homologues in an array of chelicerate models. Polarizing these dynamics using data from insect, crustacean, myriapod, onychophoran and tardigrade counterparts, we show that the acquisition of a tarsal selector function for *cll* evolved in the last common ancestor of Chelicerata, highlighting a case of developmental systems drift (i.e. the structure remains homologous, but the underlying genetic mechanisms patterning the structure have shifted over evolutionary time) in arthropod appendage patterning.

## 2. Material and Methods

### (a) Bioinformatics and orthology inference

Homologues of *cll* and *al* were identified in the genomes of the chelicerates *P. opilio* (Opiliones) [26], *Parasteatoda tepidariorum* (Araneae) [27], *Ixodes scapularis* (Parasitiformes) [28], *Archegozetes longisetosus* (Acariformes) [29] and *P. litorale* (Pycnogonida) [30]. As comparative data, we also identified two homologues of *cll* in the genome of the crustacean *Parhyale hawaiensis* [31]. As queries, we used the peptide sequences of *cll* and *al* from the velvet worm *Euperipatoides kanangrensis* (GenBank accession nos. CDK60407.1; CDK60408.1) and performed tBLASTn searches against the annotated coding sequence files of each genome assembly, retaining hits with e-value <1 × 10^−20^. The homologue of *P. opilio Delta* was additionally queried from the harvestman genome using peptide sequences from *D. melanogaster* (NCBI reference sequence NM_057916.4) and tBLASTn searches as described above. The identities of all retained hits were validated using SMARTBLAST prior to gene tree analysis.

Multiple sequence alignment was performed using CLUSTAL Omega [32]. Gene trees were inferred using IQ-TREE v. 3 [33] with an LG + I + G substitution model and nodal support was estimated using 10 000 ultrafast bootstrap replicates (electronic supplementary material, figures S7–S10). We performed two sets of analyses. First, we included all *cll* and *al* homologues in a 10-taxon alignment, using as outgroups all *D. melanogaster* and *P. opilio* homeobox sequences previously identified in a recent comprehensive analysis of chelicerate homeobox genes, which previously identified both *cll* and *al* in *P. opilio* [23] (electronic supplementary material, figure S7). This alignment was trimmed to retain only the homeobox domain, following the published analysis. Next, to explore relationships between paralogues of both genes, we generated separate alignments using full-length sequences for both *cll* and *al* (electronic supplementary material, figures S8 and S9). Gene trees were rooted using closely related genes from the *D. melanogaster* and *P. opilio* proteomes (*BarH1* for *cll; Pph13* for *al*). Similarly, alignment of *Delta* sequences was performed using the closely related gene *Serrate* for rooting the gene tree (electronic supplementary material, figure S10). Alignments are provided in electronic supplementary material, files S1–S4.

Other *P. opilio* genes in this study (*Distal-less*, *Serrate* and *odd-skipped*) were identified in previous works [16,24].

### (b) Gene expression assays

Embryos were fixed and assayed for fluorescent detection of gene expression following established protocols, as detailed previously for *P. hawaiensis* and chelicerate species [16,26,34]. For *I. scapularis* culture, all procedures involving animal subjects were approved by the Institutional Animal Care and Use Committee (IACUC) at the University of Nevada-Reno (IACUC #21–01-1118–1). In species other than *A. longisetosus*, we targeted selected developmental stages where podomeres could be individually recognized.

Probe design for hybridization chain reaction (HCR) consisted of 6–28 probe pairs, depending upon the length of the available template sequence. Probes were designed with the HCR Probe Maker tool [35]. Output probe sequences for all species are provided in electronic supplementary material (tables S1–S13).

### (c) RNA interference

Primer pairs designed for cloning *Po-cll* are available in electronic supplementary material (table S14). Fragments of *Po-cll* were amplified using standard PCR protocols and cloned using a TOPO TA Cloning Kit using One Shot Top10 chemically competent *Escherichia coli* (ThermoFisher) following the manufacturer’s protocol, and PCR product identity was verified via Sanger sequencing with M13 universal primers. Double-stranded RNA (dsRNA) was synthesized following the manufacturer’s protocol using a MEGAscript T7 kit (Ambion/Life Technologies) from amplified PCR product. The quality of dsRNA was assessed and concentrations adjusted using a NanoDrop ONE to 3.8–4.2 μg/μl. dsRNA was mixed with vital dyes for visualization of injections. Microinjection under halocarbon-700 oil (Sigma-Aldrich) was performed as previously described [36], targeting stage 8 (i.e. the appearance of limb buds) embryos. Negative control embryos were injected with distilled water mixed with vital dyes under identical conditions. Subsets of developing embryos were fixed for HCR; the remainder were developed at 26°C until hatching.

### (d) Imaging

Brightfield microscopy was performed using a Nikon SMZ fluorescence stereomicroscope mounted with a DSFi2 digital colour camera driven by Nikon Elements software. Images of *P. opilio* appendages were captured at varying focal planes and compiled into focused stacks with Helicon Focus v. 6.6.1. Confocal laser scanning microscopy was performed using a Zeiss LSM 710, Zeiss LSM 880 or Zeiss LSM 980 microscope driven by Zen software.

For *P. litorale*, confocal laser scanning microscopy was performed with a Leica SP5 microscope, driven by Leica Application Suite-Advanced Fluorescence (LAS-AF) software. Beyond the documentation of gene expression (594 nm and 633 nm laser lines) and DAPI counterstain (405 nm laser line), cuticular autofluorescence was separately recorded with the 488 nm laser line. Using the software Amira 3D (version 2021.1; ThermoFisher Scientific), the cuticular signal in the 488 nm channel was semi-automatically segmented (grey-value-based thresholding), and the voxels included in the resulting material were set to grey value 0 in all other channels via the ‘Arithmetic’ function to separate cuticular autofluorescence from gene expression signals.

## 3. Results and Discussion

### (a) *cll* is required for the patterning of the harvestman tarsus, but not the claw

**Figure 2.**
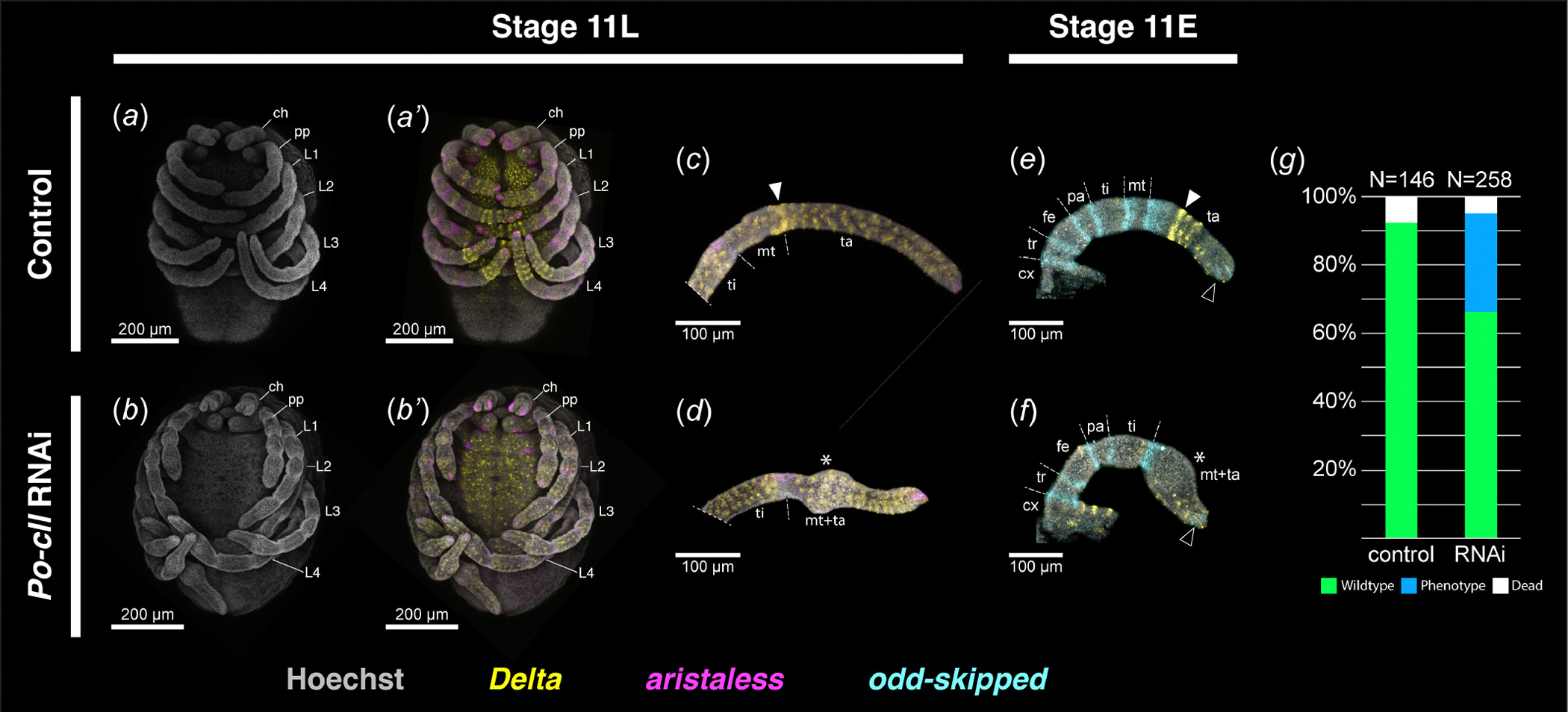
Knockdown of *Po-cll* disrupts tarsomere patterning mechanisms. (*a, b*) Late stage 11 embryos with isolated nuclear counterstaining (grey). (*a’–d*) Multiplexed visualization of nuclear counterstaining, and expression of *Po-Dl* (yellow) and *Po-al* (magenta) in control and *Po-cll* RNAi treatments. (*a’*) Same embryo as in (*a*). (*b’*) Same embryo as in (*b*). (*c*) Leg II of negative control embryo. (*d*) Leg II of *Po-cll* RNAi embryo. (*e*, *f*) Leg II of early stage 11 control (*e*) and RNAi embryos (*f*) with multiplexed expression of *Po-Dl* and *Po-odd* (cyan). Note the strong ring of *Po-Dl* at the metatarsus-tarsus boundary in control embryos (white arrowheads) and absence of the *Po-Dl* ring (asterisk) in RNAi embryos. Expression of *Po-odd* is retained in the putative pretarsus in RNAi embryos (black arrowheads). (*g*) Distribution of phenotypic outcomes in negative control and *Po-cll* RNAi experiments. Abbreviations as in figure 1.

**Figure 3.**
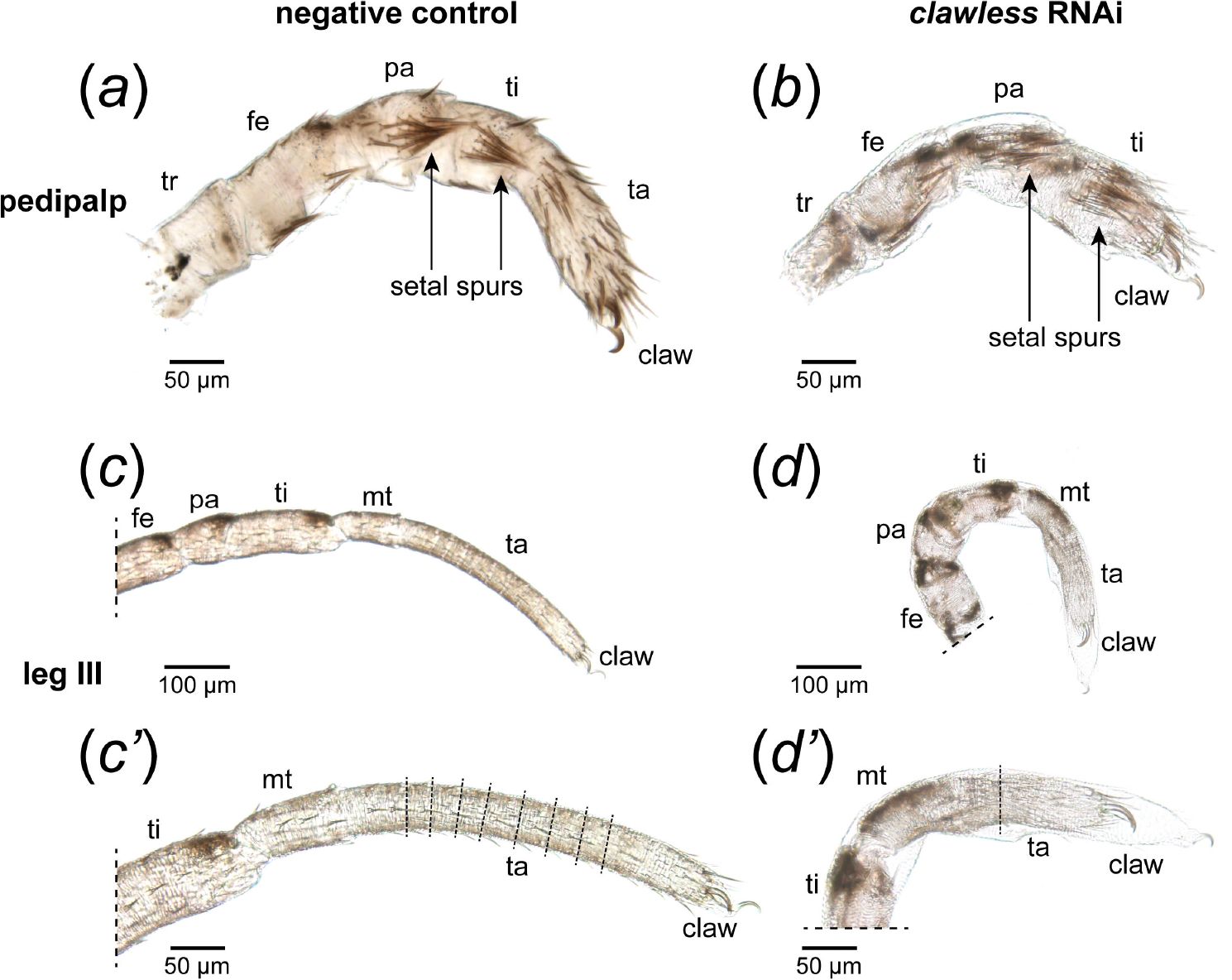
*Po-cll* is necessary for the development of the tarsus, but not the claw, in *P. opilio*. (*a–d*) Appendage mounts of hatchlings in lateral view. (*a*) Pedipalp of negative control hatchling. (*b*) Pedipalp of *Po-cll* RNAi hatchling. (*c*) Leg III of negative control hatchling. (*c’*) Magnification of tarsus III, showing tarsomere boundaries (dashed lines). (*d*) Leg III of *Po-cll* RNAi hatchling. (*d’*) Magnification of tarsus III in *Po-cll* RNAi hatchling; note truncation of the tarsus and absence of comparable tarsomere boundaries. Abbreviations as in figure 1.

Consistent with the late localization of *Po-cll* expression to the metatarsus-tarsus boundary, *Po-cll* RNA interference (RNAi) yielded phenotypes exhibiting fusion of the metatarsus and tarsus (figure 2). Assays of *Po-cll* in RNAi embryos confirmed on-target depletion in embryos exhibiting tarsal defects (*n* = 18/18; electronic supplementary material, figure S1). To validate the interpretation of segmental fusion, a subset of RNAi embryos was assayed for the expression of *odd-skipped* (*odd*), a marker of developing podomere boundaries in arthropod appendages [24,37,38]. Whereas the appendages of negative control embryos exhibited six ring domains of *odd* at podomere boundaries, RNAi embryos exhibited only five such domains, with a missing *odd* domain corresponding to the boundary of metatarsus and tarsus (figure 2e,f). Notably, faint *odd* expression is retained at the distal terminus of the appendages in both control and RNAi embryos, suggesting no defects of the pretarsus boundary (figure 2e,f). Following embryogenesis, 28.7% (*n* = 74/258) of hatchlings exhibited the fusion phenotype, with slight variations in the resulting deformation (figures 2 and 3; electronic supplementary material, figure S2). Mild phenotypes were characterized by an outward bulging of the tissue at the presumptive malformed segmental boundary during embryonic stages (figure 2b–b’,d,f), whereas severe phenotypes were characterized instead by distal appendages bearing a pronounced bent or kinked appearance, which we attribute to asymmetric projections of tissue at the malformed joint (electronic supplementary material, figure S2). The severe phenotype, however, never impacted all appendages in a single embryo; one or two appendages bearing the kinked appearance were common. Both phenotypic classes, however, are characteristic of podomeric fusions following RNAi against limb-patterning transcription factors in both arachnid and insect exemplars [16,39–41]. No *Po-cll* RNAi hatchlings exhibited defects or absence of the tarsal claws or of cheliceral tips (figure 3b,d–d’; *contra* the claws and arista of *D. melanogaster* [15]).

Coincident with the segmental fusion was a significant shortening of the tarsus in *Po-cll* RNAi hatchlings (figure 3). The effect of *Po-cll* RNAi on the tarsus is reminiscent of failed tarsomere formation in knockdowns of *EgfrA* [26]. In insects, simultaneous formation of tarsomeres is achieved by establishment of a distal-to-proximal gradient of EGFR activity, which provides positional information required for the precise activation of transcription factors like *dachshund, bric-a-brac* and *Bar*; these genes are responsible for subdivision of the tarsus into proximal, medial and distal domains, respectively. Regulatory interactions amongst these and other transcription factors result in unique gene expression combinations in each of the five tarsomeres of *D. melanogaster*. Thereafter, heterogeneous expression of *Notch* and its ligands *Delta* and *Serrate* promote regions of cell proliferation via activation of downstream targets like *AP-2,* and regions of apoptotic activity via activation of targets *reaper* and *head involution defective*, resulting in the formation of tarsomere boundaries [11,19,42]. By contrast, in *P. opilio*, tarsomeres are not formed simultaneously, but rather sequentially as the tarsus elongates in later stages of development. To characterize the nature of tarsomere patterning in *P. opilio*, we first investigated the expression dynamics of *Po-Delta* (*Po-Dl*) and *Po-Serrate* (*Po-Ser*) in the elongating tarsus.

In stage 9 embryos, *Po-Ser* is expressed in ring domains corresponding to putative segmental boundaries, including the metatarsal-tarsal boundary (electronic supplementary material, figure S3a). Expression in this stage is strongest in the proximal boundaries and is only weakly detected in distal appendage territories. In later stages, *Po-Ser* remains localized to segmental boundaries, but does not exhibit additional ring domains in the elongating tarsus, likely refuting a direct role in tarsomere formation (electronic supplementary material, figure S3b–d). Tarsal expression of *Po-Ser* is diffuse and patchy, likely coincident with cells fated for sensory or neural fates given previously characterized roles for Notch signalling in the regulation of proneural genes including the *achaete-scute* and *Enhancer of split* complexes [43,44].

*Po-Dl* likewise localizes to putative segmental boundaries during early stages of appendage elongation, again including the metatarsus-tarsus boundary. Yet by stage 9, an additional stronger domain of *Po-Dl* expression is detected in the leg II tarsus, distal to the segmental boundary of *Po-Ser* expression (electronic supplementary material, figure S3a). In progressively later stages, the same ring domain appears in legs I, III and IV. In these stages, the initially singular domain in each appendage multiplies, with sequential addition of ring domains distally (electronic supplementary material, figure S3b–g). Given the sequential appearance of these domains, as well as the canonical role of *Dl* in patterning segmental boundaries, *Po-Dl* appears to be a reliable readout of sequential tarsomere formation in *P. opilio*. Consistent with this interpretation, such ring domains are absent in the pedipalpal tarsus, which does not develop tarsomeres. Earlier onset of tarsal expression in leg II likewise coincides with observations of higher tarsomere counts in leg II, owing to its antenniform morphology (elongation of podomeres; concentration of sensory organs), analogous to the antenniform appendages of lineages such as Amblypygi and Thelyphonida.

In *Po-cll* RNAi embryos, no ring domain of *Po-Dl is* detected in the metatarsal-tarsal boundary, and no additional ring domains are observed in the tarsus (figure 2c–f). Distal expression is restricted to putative sensory structures. The disruption of *Po-Dl* expression dynamics in this region is strongly suggestive of failed tarsomere formation.

Both truncations in tarsus length and failed tarsomere formation were observed in hatchlings following RNAi treatment (figure 3). This truncation is most dramatically observed in the pedipalp, an appendage that is distinguished from the walking legs by the lack of a metatarsus. In the pedipalps of wild-type hatchlings, two prominent setal tufts (‘spurs’) occur in the distal territories of the patella and tibia (figure 3a). In *Po-cll* RNAi hatchlings, however, the tibial spur appears distally adjacent to the claws, suggesting near-complete absence of the tarsus, without affecting the tibia (figure 3b). This result supports the interpretation that *Po-cll* is required specifically for maintenance of the tarsal segment in both the leg and the pedipalp. Likewise, whereas tarsomere boundaries are apparent in hatchling walking legs, RNAi hatchlings exhibit drastic reductions in the number of correctly formed tarsomeres (figure 3c–d’).

Taken together, these results support a novel role for *Po-cll* in the patterning of the tarsus, rather than the distalmost territory (claws; aristae) as in other arthropod lineages. The impact of this functional change on other members of the tarsal-patterning gene regulatory network is a logical next step for investigation in *P. opilio*. While we trialled RNAi against *Po-al*, these experiments did not elicit any defect in the tarsal claws or cheliceral tips (data not shown).

### (b) A broad tarsal domain of *cll* is phylotypic of Chelicerata

To pinpoint the phylogenetic origin of these expression dynamics, we surveyed the expression of *cll* in phylogenetically significant representatives of Chelicerata, including the sister group to the remaining chelicerates (the sea spiders). Where available, *al* orthologues were also surveyed to test the association of *al* expression with the claw.

**Figure 4.**
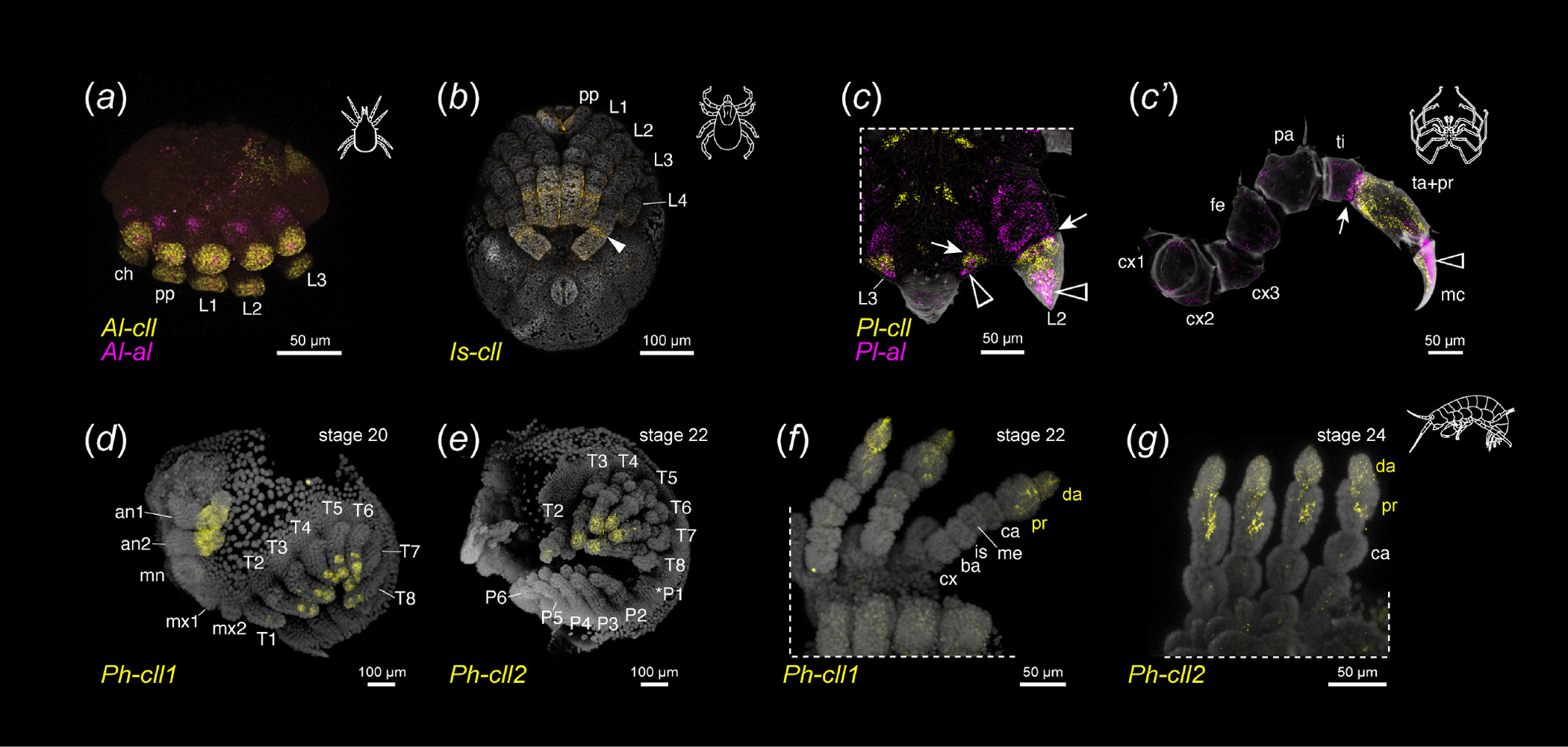
Proximal expansion of *cll* expression beyond the distal terminus is phylotypic of Chelicerata. (*a*) Multiplexed expression of *cll* (yellow) and *al* (magenta) in the acariform mite *A. longisetosus*. (*b*) Stage 13 embryo of the parasitiform tick *I. scapularis* with expression of *cll*. White arrowhead: basi- and telotarsus segmental boundary. (*c*) Expression of *Pl-cll* and *Pl-al* with cuticular autofluorescence (grey) in the sea spider *P. litorale*. (*d*) Ventral view of instar IV posterior body pole and leg 2 limb bud. (*c’*) Leg 1 of advanced instar V. Black arrowhead: dorsal *Pl-al* domain in the distal limb bud tips and in the main claw of legs. Arrow: proximal boundary of *Pl-cll* expression in limb buds and region of *Pl-cll* and *Pl-al* co-expression at the tibial-tarsal boundary in developing legs. (*d–g*) Expression of two *cll* paralogues in the amphipod *P*. *hawaiensis*. (*d*) *Ph-cll1* expression in a stage 20 embryo. (*e*) *Ph-cll2* expression in a stage 22 embryo. Asterisk denotes damaged P1 appendage following dissection. (*f*) Detail of *Ph-cll1* expression in thoracopods of a stage 22 embryo. (*g*) Detail of *Ph-cll2* expression in thoracopods of a stage 24 embryo. Abbreviations: an, antenna; ba, basis; ca, carpus; cx, coxa; da, dactylus; is, ischium; mc, main claw; me, merus; mn, mandible; mx, maxilla; P, pleopod; pr, propodus; T, thoracopod; others as in figure 1.

In the acariform mite, *A. longisetosus* (Acariformes: Sarcoptiformes), *Al-cll* expression appears early in the appendage primordia (electronic supplementary material, figure S4a–a”’), localizing in limb bud stages to a cap-like distal domain (figure 4a, electronic supplementary material, figure S4b–e”’). Unlike the other surveyed taxa, however, *Al-cll* does not exhibit heterogeneous intensity; no clear ring domains were observed. The onset of *Al-al* expression occurs only after initial outgrowth of the limb buds from the body wall (electronic supplementary material, figure S4b–b”’). *Al-al* is expressed in two distinct domains, one as a diffuse domain in the proximal territory of all five appendage pairs of the hexapodous embryo, as well as a minute domain of several *al-*positive cells in the distal terminus within the broader domain of *Al-cll* (figure 4a, electronic supplementary material, figure S4c-e”’), comparable to expression patterns of these genes in early developmental stages in *P. opilio* [17]. Despite the lack of clear podomere boundaries in the stages examined, the extension of *Al-cll* expression proximally beyond the distal *Al-al* domain reflects the same expression dynamics observed in *P. opilio*.

Embryos of the parasitiform tick, *I. scapularis* (Parasitiformes: Ixodida), were surveyed for *Is-cll* expression at stage 13, wherein podomere boundaries are visible in the first three pairs of walking legs; only the distal tip of the fourth pair of legs are visible during retraction and formation of the hexapodous larva [45]. *I. scapularis* embryos were also surveyed for *Is-al,* but expression was not detected in the stages surveyed (data not shown). *Is-cll* is expressed broadly throughout the distal appendage territories of the walking legs at stage 13, including weakly in the remaining leg IV bud (figure 4b). As in the harvestman, however, expression in the legs is heterogeneous; the highest intensity of expression localizes to the putative boundary of basi- and telotarsus. This pattern is suggestive of shared distal appendage patterning dynamics with the harvestman. Likewise, this expression domain is consistent with homology of the tick basitarsus and harvestman metatarsus, supporting the interchangeable use of both metatarsus and basitarsus by some authors [46,47].

Spiders, as members of Arachnopulmonata (a group of six chelicerate orders ancestrally bearing book lungs), bear a whole genome duplication and thus two copies of *cll* [27]. The expression of both *cll* paralogues has been examined previously in embryos of both *P. tepidariorum* (Entelegynae) and *Pholcus phalangioides* (Haplogynae) [48]. In both species, despite differences in expression patterns, the two *cll* paralogues broadly exhibit comparable dynamics. Each paralogue is expressed first in a broad, distal appendage domain in early stages of development, encompassing the putative pretarsus. In later stages, each refines into a heterogeneous domain, forming stronger ring domains within regions of broad, diffuse expression at putative distal podomere boundaries. Crucially, however, in these later stages, expression does extend to the distal terminus of the appendages, except for *P. phalangioides cll-1,* which exhibits a single strong domain localized to a minute spot in the ventral compartment of the distalmost appendage territory. These expression patterns were corroborated by a subsequent survey of *cll* paralogues in *P. tepidariorum*, albeit with a smaller range of surveyed stages wherein podomere boundaries were not identifiable [23].

The sea spider *P. litorale* hatches as a protonymphon larva with only three appendage pairs: the chelicerae and two pairs of larval appendages (mostly resorbed in adulthood in Pycnogonidae). The walking legs I–IV are added sequentially alongside the remaining body segments during successive moults. We previously showed that *Pl-cll* is strongly associated with two distal podomeres (the tarsus and propodus), but the exact homology of the sea spider claw (sometimes termed dactylus) is an outstanding question and impedes unambiguous comparison [17]. To surmount this ambiguity, we examined postembryonic instars for co-expression of *Pl-cll* and *Pl-al*. In instar II, both *Pl-cll* and *Pl-al* are detected in the leg I primordium that flanks the posterior body pole. Both genes are partially co-expressed at the distalmost area of the primordium; the more restricted *Pl-al* territory is embedded in the wider cap-like *Pl-cll* expression domain (electronic supplementary material, figure S5a). By instar III, when the leg I bud has begun outgrowth from the body wall, *Pl-al* is weakly expressed in a wide domain in the proximal limb bud, as well as strongly at the distal terminus (electronic supplementary material, figures S5b,c and S6a). *Pl-cll* expression occupies the intervening medial appendage domain, with a sharp proximal border at the boundary of *Pl-al* expression. At the limb bud’s distal terminus, the *Pl-cll* domain extends into the ventral compartment of the developing claw, complementary to the dorsally concentrated *Pl-al* territory (figure 4d, electronic supplementary material, figure S6a, a’, b’). In instars IV and V, wherein the first functional podomeres have formed in legs I and II, respectively, *Pl-cll* is expressed in the precursor podomere that will eventually divide into mature tarsus and propodus, and the ventral compartment of the claw (figure 4d’, electronic supplementary material, figure S6c–d’). *Pl-al* is detected diffusely in all further proximal podomeres, as well as strongly in the dorsal compartment of the claw (electronic supplementary material, figure S6c–d’). Both genes exhibit little to no co-expression, with the exception of a narrow ring of elevated *Pl-al* expression at the proximal boundary of the *Pl-cll* domain (electronic supplementary material, figure S6d, d’). The short tarsus will differentiate in this co-expression domain, which becomes more distinct as the growing leg tissues condense prior to the moults. Once the tarsus and propodus are fully separated (instar VI), the ring of *Pl-cll* and *Pl-al* co-expression becomes more diffuse; only a small area with distinct expression overlap persists in the dorsal joint of the tarsus (electronic supplementary material, figure S6e, e’). By contrast, the claw retains its dorsal *Pl-al* and ventral *Pl-cll* domains (electronic supplementary material, figure S6e,e’). These results support the interpretation that the sea spider tarsus, propodus and claw are homologues of the arachnid metatarsus, tarsus and pretarsus, respectively. This reconstruction suggests that the additional (auxiliary) claws observed in some sea spider species may represent further derivations of the pretarsus, comparable to accessory claws (e.g. spider silk hooks) or pseudonychia of arthropods.

**Figure 5.**
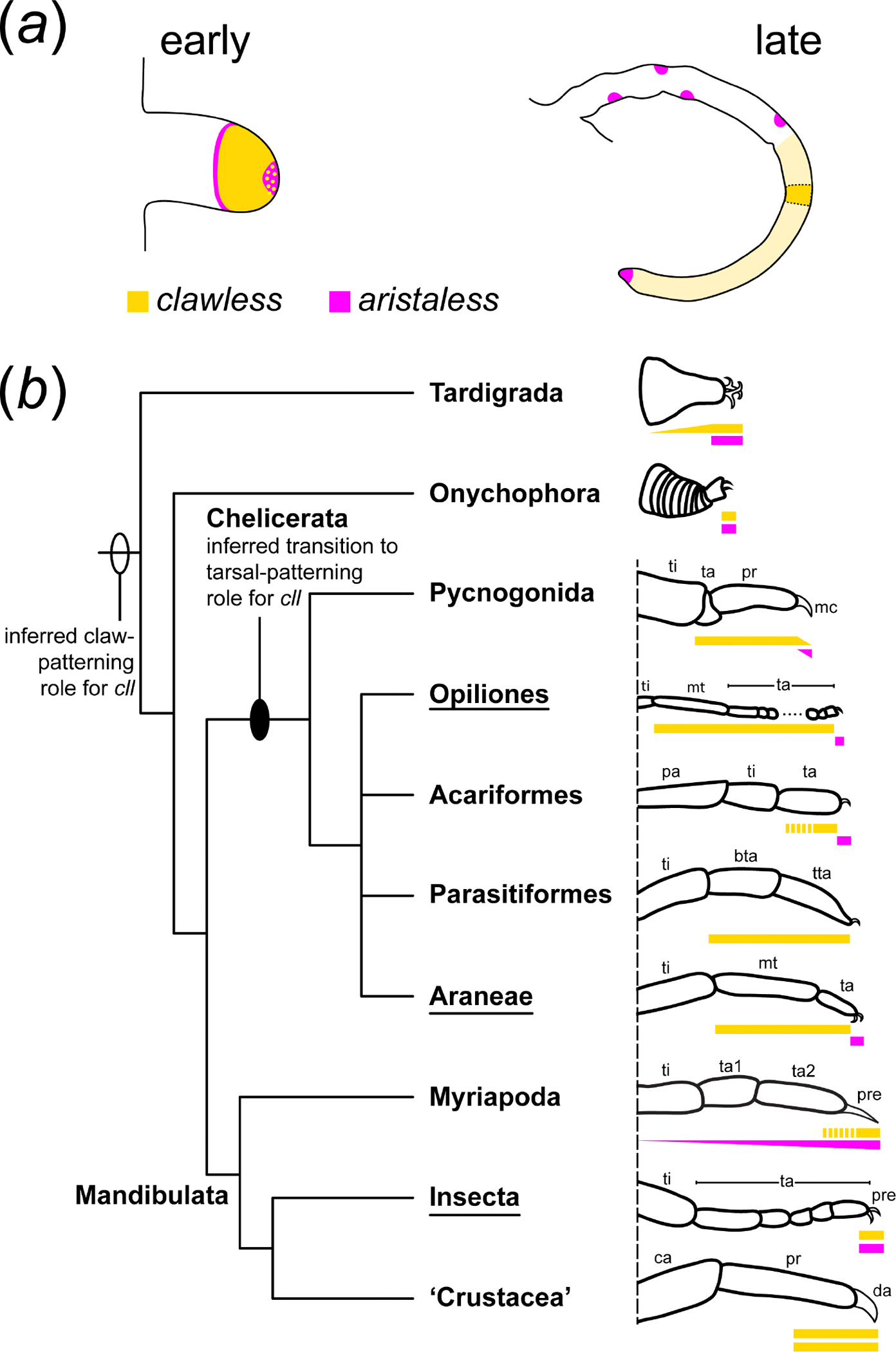
Developmental system drift in distal appendage patterning dynamics in Panarthropoda. (*a*) Simplified representations of expression dynamics of *cll* and *al* in the appendages of *P. opilio*. (*b*) Schematic representations of distal podomere morphology and *cll* and *al* dynamics in representative lineages of Panarthropoda (left), with reconstruction of inferred ancestral states at major nodes, based on a combination of available expression and functional data. Underlined taxa indicate lineages for which functional data are available. Additional proximal domains of *al* are not shown. Note restricted distal domains of *cll* expression in Onychophora and Mandibulata. Proximal *al* domains have been excluded for simplicity. Triangular bars represent expression restricted to dorsal or ventral compartments of the proximo-distal appendage axis. Stippled bars for *cll* expression in the acariform and myriapod exemplars reflect uncertainty in the extent of this domain due to lack of sampling of older stages [5]. Abbreviations: bta, basitarsus; tta, telotarsus; pre, pretarsus; others as in figures 1 and 4.

Taken together, these data support the interpretation that a broad *cll* domain subtending the pretarsus (marked by *al*) is phylotypic for Chelicerata, and that a new role for *cll* as a tarsal selector may have evolved in the most recent common ancestor of chelicerates (figure 5). While functional data for chelicerate homologues of *cll* remain limited, this inference is supported in part by RNAi experiments targeting *cll-1* in the spider *P. tepidariorium* [48]. In that work, mild RNAi phenotypes exhibited comparable fusions of metatarsus and tarsus, coincident with similar protrusions of tissue or kinked appearances at the malformed joint. Severe RNAi phenotypes in this species exhibited total loss of the tarsal segment. No defects to the tarsal claws were ever reported. Thus, the spectrum of RNAi phenotypes in the spider overlaps substantially with its *P. opilio* counterpart.

### (c) *cll* expression dynamics across Panarthropoda are consistent with developmental system drift in Chelicerata

Expression and functional dynamics of chelicerate *cll* homologues stand in opposition to the conserved *cll* dynamics seen in the mandibulate arthropods. In the holometabolous insect models *D. melanogaster* and *T. castaneum*, *cll* expression is restricted to the centre of the imaginal disc or the pretarsus of the leg, respectively [7,15,21]. Likewise, in the pill millipede *Glomeris marginata*, *cll* is again restricted to the distal terminus of the developing walking legs [5]. To augment this taxonomic sampling, we surveyed expression of two *cll* homologues in the amphipod crustacean *P. hawaiensis*. Gene tree analysis recovered these two copies as most closely related, suggesting a lineage-specific duplication within some subset of crustaceans (electronic supplementary material, figure S7). We found that the two copies had similar expression patterns; both were expressed in the distal territory of both antennal pairs, the T1–T8 appendages, and in small domains in the central nervous system (figure 4d,e). Neither copy was expressed in the mandible, maxillae or pleopods. However, *Ph-cll1* was expressed in two discrete patches during leg segmentation stages, which resolved into the distal propodus and dactylus by stage 23 (figure 4f). By contrast, *Ph-cll2* was more homogeneously expressed in the distal territory of the T1–T8 appendages. By stage 24, the strongest expression of *Ph-cll2* also corresponded to the distal propodus and dactylus (figure 4e).

The dynamics observed across Mandibulata are also characteristic of the sister groups of Arthropoda. In the lobopods of onychophorans, the sister group to the arthropods, *cll* is expressed in the distal tip, coincident with the onset of *al* expression in the same territory [49]. The lobopods of tardigrades also bear distal expression of *cll* [50]. In this lineage, *cll* expression is also detected proximal to the distal tip, extending to the medial appendage territory. However, unlike the proximal expansion of *cll* in chelicerates, proximal tardigrade *cll* is only detected in the posterior compartment of the appendage. The absence of *cll* expression at stage 17, wherein the claws are fully formed, is suggestive of a primary role in claw formation. Unlike other panarthropods, tardigrade *al* expression dynamics seem to be unique. While expressed in the distal appendage of the tardigrade, *al* is restricted only to an internal compartment, absent from the distal ectoderm, with only limited co-expression with *cll* at the margin of the ectoderm [50].

The sum of these available datasets suggests that retention of *cll* expression in the distal tip of the developing appendages across Panarthropoda reflects an ancestral role in the patterning and formation of the claws. However, this function was likely lost in the chelicerate common ancestor following the split from Mandibulata, with no observable impact on the expression of the phenotype. This event is consistent with a case of developmental system drift and highlights the lability (tendency to shift or change) of the gene regulatory network underlying the patterning of the proximo-distal appendage axis across panarthropods. Several cases of labile gene expression have been observed in the arthropod appendage literature, as exemplified by surveys of *nubbin*, *homothorax*, *exd*, *dachshund*, *Lim1*, *Sp6–9* and even *Distal-less* [6,12,14,16,51–56]. The variability in expression domains of these transcription factors even within a subset of arthropods (e.g. insects; arachnids) suggests that the patterning of the leg axis and its subdivisions is robust, possibly by way of redundant wiring of the underlying gene network [57]. Functional evidence for this redundancy is limited, though a recent example supporting its persistence is the redundancy of *exd* and *dachshund-2* function in patterning the distal segmental boundary of the patella in a harvestman and a spider, respectively [16,40].

More generally, this case study underscores the value of comparative data and enriching taxonomic sampling, even for clades with well-established model systems, towards closing gaps in the understanding of morphological evolution. With respect to chelicerates, two remaining taxon-specific innovations whose genetic underpinnings are still not understood include the three coxae of sea spiders (recently proposed to be subdivisions of the coxa of arachnids, based on the expression of *SoxNeuro* homologues [17]) and the metatarsus (or sea spider ‘tarsus’), whose presence distinguishes the walking leg of most arachnids from the pedipalp.

## Supplementary Material

Supplementary materials2

Supplementary materilas 3

Supplementary material 1

Supplementary materials 4

Supplementary data

Electronic supplementary material is available online at https://doi.org/10.6084/m9.figshare.c.8225229.

## Data Availability

All raw files of confocal microscopy hyperstacks (.czi; .lif) are available from the Dryad Digital Repository: [58]. Supplementary material is available online [59].

## References

[1] Jockusch EL, Williams TA, Nagy LM. 2004 The evolution of patterning of serially homologous appendages in insects. Dev. Genes Evol. 214, 324–338. (doi:10.1007/s00427-004-0412-6) 15170569

[2] Popadíc A, Panganiban G, Rusch D, Shear WA, Kaufman TC. 1998 Molecular evidence for the gnathobasic derivation of arthropod mandibles and for the appendicular origin of the labrum and other structures. Dev. Genes Evol. 208, 142–150. (doi:10.1007/s004270050165) 9601987

[3] Dong PDS, Chu J, Panganiban G. 2001 Proximodistal domain specification and interactions in developing *Drosophila* appendages. Development 128, 2365–2372. (doi:10.1242/dev.128.12.2365) 11493555

[4] Beermann A, Jay DG, Beeman RW, Hülskamp M, Tautz D, Jürgens G. 2001 The short antennae gene of *Tribolium* is required for limb development and encodes the orthologue of the *Drosophila* Distal-less protein. Development 128, 287–297. (doi:10.1242/dev.128.2.287) 11124123

[5] Janssen R 2017 Gene expression reveals evidence for EGFR-dependent proximal-distal limb patterning in a myriapod. Evol. Dev. 19, 124–135. (doi:10.1111/ede.12222) 28444830

[6] Setton EVW, Sharma PP. 2018 Cooption of an appendage-patterning gene cassette in the head segmentation of arachnids. Proc. Natl Acad. Sci. USA 115, E3491–E3500. (doi:10.1073/pnas.1720193115) 29581309 PMC5899462

[7] Grossmann D, Prpic NM. 2012 Egfr signaling regulates distal as well as medial fate in the embryonic leg of *Tribolium castaneum*. Dev. Biol. 370, 264–272. (doi:10.1016/j.ydbio.2012.08.005) 22921411

[8] Abzhanov A, Kaufman TC. 2000 Homologs of *Drosophila* appendage genes in the patterning of arthropod limbs. Dev. Biol. 227, 673–689. (doi:10.1006/dbio.2000.9904) 11071783

[9] Pechmann M, Prpic NM. 2009 Appendage patterning in the South American bird spider Acanthoscurria geniculata (Araneae: Mygalomorphae). Dev. Genes Evol. 219, 189–198. (doi: 10.1007/s00427-009-0279-7) 19266215

[10] Prpic NM, Damen WGM. 2009 Notch-mediated segmentation of the appendages is a molecular phylotypic trait of the arthropods. Dev. Biol. 326, 262–271. (doi:10.1016/j.ydbio.2008.10.049) 19046962

[11] Natori K, Tajiri R, Furukawa S, Kojima T. 2012 Progressive tarsal patterning in the *Drosophila* by temporally dynamic regulation of transcription factor genes. Dev. Biol. 361, 450–462. (doi:10.1016/j.ydbio.2011.10.031) 22079694

[12] Turchyn N, Chesebro J, Hrycaj S, Couso JP, Popadić A. 2011 Evolution of nubbin function in hemimetabolous and holometabolous insect appendages. Dev. Biol. 357, 83–95. (doi:10.1016/j.ydbio.2011.06.014) 21708143 PMC3178182

[13] Bruce HS. 2021 How to align arthropod legs. bioRxiv (doi:10.1101/2021.01.20.427514)

[14] Tsuji T, Sato A, Hiratani I, Taira M, Saigo K, Kojima T. 2000 Requirements of Lim1, a *Drosophila* LIM-homeobox gene, for normal leg and antennal development. Development 127, 4315–4323. (doi:10.1242/dev.127.20.4315) 11003832

[15] Kojima T, Tsuji T, Saigo K. 2005 A concerted action of a paired-type homeobox gene, aristaless, and a homolog of Hox11/tlx homeobox gene, clawless, is essential for the distal tip development of the *Drosophila* leg. Dev. Biol. 279, 434–445. (doi:10.1016/j.ydbio.2004.12.005) 15733670

[16] Klementz BC 2024 A novel expression domain of extradenticle underlies the evolutionary developmental origin of the chelicerate patella. Mol. Biol. Evol. 41, msae188. (doi:10.1093/molbev/msae188) 39235104 PMC11422720

[17] Klementz BC, Brenneis G, Laumer EM, Neu SM, Harvey MS, Sharma PP. 2025 Evolution and homology of leg segments in chelicerata: evo-devo solutions to century-old challenges. Arthropod Struct. Dev. 87, 101446. (doi:10.1016/j.asd.2025.101446) 40311600

[18] Tajiri R, Tsuji T, Ueda R, Saigo K, Kojima T. 2007 Fate determination of *Drosophila* leg distal regions by trachealess and tango through repression and stimulation, respectively, of Bar homeobox gene expression in the future pretarsus and tarsus. Dev. Biol. 303, 461–473. (doi:10.1016/j.ydbio.2006.11.026) 17187773

[19] Kojima T 2017 Developmental mechanism of the tarsus in insect legs. Curr. Opin. Insect Sci. 19, 36–42. (doi:10.1016/j.cois.2016.11.002) 28521941

[20] Pueyo JI, Galindo MI, Bishop SA, Couso JP. 2000 Proximal-distal leg development in *Drosophila* requires the *apterous* gene and the *Lim1* homologue *dlim1*. Development 127, 5391–5402. (doi:10.1242/dev.127.24.5391) 11076760

[21] Campbell G 2005 Regulation of gene expression in the distal region of the *Drosophila* leg by the Hox11 homolog, C15. Dev. Biol. 278, 607–618. (doi:10.1016/j.ydbio.2004.12.009) 15680373

[22] Miyazono K, Zhi Y, Takamura Y, Nagata K, Saigo K, Kojima T, Tanokura M. 2010 Cooperative DNA-binding and sequence-recognition mechanism of aristaless and clawless. EMBO J. 29, 1613–1623. (doi:10.1038/emboj.2010.53) 20389279 PMC2876955

[23] Aase-Remedios ME, Janssen R, Leite DJ, Sumner-Rooney L, McGregor AP. 2023 Evolution of the spider homeobox gene repertoire by tandem and whole genome duplication. Mol. Biol. Evol. 40, msad239. (doi:10.1093/molbev/msad239) 37935059 PMC10726417

[24] Medina-Jiménez BI, Budd GE, Janssen R. 2025 Single-cell sequencing reveals potential novel insights into appendage-patterning and joint-development in a spider. *Dev. Dyn*. In press. (doi:10.1002/dvdy.70069) PMC13353597 40772585

[25] Wolff JO, Seiter M, Gorb SN. 2015 Functional anatomy of the pretarsus in whip spiders (Arachnida, Amblypygi). Arthropod Struct. Dev. 44, 524–540. (doi:10.1016/j.asd.2015.08.014) 26386460

[26] Gainett G, González VL, Ballesteros JA, Setton EVW, Baker CM, Barolo Gargiulo L, Santibáñez-López CE, Coddington JA, Sharma PP. 2021 The genome of a daddy-long-legs (Opiliones) illuminates the evolution of arachnid appendages. Proc. R. Soc. B 288, 20211168. (doi:10.1098/rspb.2021.1168) PMC8334856 34344178

[27] Schwager EE 2017 The house spider genome reveals an ancient whole-genome duplication during arachnid evolution. BMC Biol. 15, 62. (doi:10.1186/s12915-017-0399-x) 28756775 PMC5535294

[28] Nuss AB 2023 The highly improved genome of *Ixodes scapularis* with X and Y pseudochromosomes. Life Sci. Alliance 6, e202302109. (doi:10.26508/lsa.202302109) 37813487 PMC10561763

[29] Brückner A, Barnett AA, Bhat P, Antoshechkin IA, Kitchen SA. 2022 Molecular evolutionary trends and biosynthesis pathways in the *Oribatida* revealed by the genome of *Archegozetes longisetosus*. Acarologia 62, 532–573. (doi:10.24349/pjye-gkeo)

[30] Papadopoulos N, Kulkarni SS, Baranyi C, Fromm B, Setton EVW, Sharma PP, Wanninger A, Brenneis G. The genome of a sea spider corroborates a shared hox cluster motif in arthropods with reduced posterior tagma. Evol. Biol 23, 196. (doi:10.1101/2024.11.20.624475) PMC12220506 40598291

[31] Kao D 2016 The genome of the crustacean *Parhyale hawaiensis*, a model for animal development, regeneration, immunity and lignocellulose digestion. eLife 5, e20062. (doi:10.7554/elife.20062) 27849518 PMC5111886

[32] Sievers F, Higgins DG. 2018 Clustal Omega for making accurate alignments of many protein sequences. Protein Sci. 27, 135–145. (doi:10.1002/pro.3290) 28884485 PMC5734385

[33] Nguyen LT, Schmidt HA, von Haeseler A, Minh BQ. 2015 IQ-TREE: a fast and effective stochastic algorithm for estimating maximum-likelihood phylogenies. Mol. Biol. Evol. 32, 268–274. (doi:10.1093/molbev/msu300) 25371430 PMC4271533

[34] S Bruce H 2021 Hybridization chain reaction (HCR) in situ protocol v1. protocols.io (doi:10.17504/protocols.io.bunznvf6)

[35] Kuehn E, Clausen DS, Null RW, Metzger BM, Willis AD, Özpolat BD. 2022 Segment number threshold determines juvenile onset of germline cluster expansion in *Platynereis dumerilii*. *J.* Exp. Zool. Part B Mol. Dev. Evol. 338, 225–240. (doi:10.1002/jez.b.23100) PMC9114164 34793615

[36] Sharma PP, Schwager EE, Giribet G, Jockusch EL, Extavour CG. 2013 Distal-less and dachshund pattern both plesiomorphic and apomorphic structures in chelicerates: RNA interference in the harvestman Phalangium opilio (Opiliones). Evol. Dev 15, 228–242. (doi:10.1111/ede.12029) 23809698

[37] Rauskolb C, Irvine KD. 1999 Notch-mediated segmentation and growth control of the *Drosophila* leg. Dev. Biol. 210, 339–350. (doi:10.1006/dbio.1999.9273) 10357895

[38] Heingård M, Turetzek N, Prpic NM, Janssen R. 2019 FoxB, a new and highly conserved key factor in arthropod dorsal–ventral (DV) limb patterning. EvoDevo 10, 28. (doi:10.1186/s13227-019-0141-6) 31728178 PMC6842170

[39] Angelini DR, Kaufman TC. 2004 Functional analyses in the hemipteran *Oncopeltus fasciatus* reveal conserved and derived aspects of appendage patterning in insects. Dev. Biol. 271, 306–321. (doi:10.1016/j.ydbio.2004.04.005) 15223336

[40] Turetzek N, Pechmann M, Schomburg C, Schneider J, Prpic NM. 2016 Neofunctionalization of a duplicate *dachshund* gene underlies the evolution of a novel leg segment in arachnids. Mol. Biol. Evol. 33, 109–121. (doi:10.1093/molbev/msv200) 26443673

[41] Thümecke S, Schröder R. 2022 The *odd-skipped* related gene *drumstick* is required for leg development in the beetle *Tribolium castaneum*. Dev. Dyn. 251, 1456–1471. (doi:10.1002/dvdy.347) 33871128

[42] Kojima T 2004 The mechanism of *Drosophila* leg development along the proximodistal axis. Dev. Growth Differ. 46, 115–129. (doi:10.1111/j.1440-169x.2004.00735.x) 15066191

[43] de Celis JF, Marí-Beffa M, García-Bellido A. 1991 Cell-autonomous role of Notch, an epidermal growth factor homologue, in sensory organ differentiation in *Drosophila*. Proc. Natl Acad. Sci. USA 88, 632–636. (doi:10.1073/pnas.88.2.632) 1899143 PMC50866

[44] Jennings B, Celis J de, Delidakis C, Preiss A, Bray S. 1995 Role of *Notch* and *achaete-scute* complex in the expression of *Enhancer of split* bHLH proteins. Development 121, 3745–3752. (doi:10.1242/dev.121.11.3745)

[45] Hinne IA 2025 Early embryonic development in the tick *Ixodes scapularis* suggests syncytial organization and cellularization before blastoderm formation. Evodevo 16, 4. (doi:10.1186/s13227-025-00240-y) 40281623 PMC12032745

[46] Evans GO. 1992 Principles of acarology. Wallingford, UK: CAB International. (doi:10.1079/9780851988221.0000)

[47] Krantz GW, Walter DE. 2009 A manual of acarology, 3rd edn. Lubbock, TX: Texas Tech University Press.

[48] Zhang N 2016 Developmental studies of appendage patterning and formation in spiders. PhD thesis, Göttingen, Germany, Georg-August University. (doi:10.53846/goediss-5988)

[49] Oliveira MB 2014 Expression of arthropod distal limb-patterning genes in the onychophoran *Euperipatoides kanangrensis*. Dev. Genes Evol. 224, 87–96. (doi:10.1007/s00427-014-0466-z) 24519327

[50] Mapalo MA, Game M, Smith FW, Ortega-Hernández J. 2024 Expression of distal limb patterning genes in *Hypsibius exemplaris* indicate regionalization and suggest distal identity of tardigrade legs. EvoDevo 15, 15. (doi:10.1186/s13227-024-00235-1) 39538290 PMC11562647

[51] Sharma PP, Tarazona OA, Lopez DH, Schwager EE, Cohn MJ, Wheeler WC, Extavour CG. 2015 A conserved genetic mechanism specifies deutocerebral appendage identity in insects and arachnids. Proc. R. Soc. B 282, 20150698. (doi:10.1098/rspb.2015.0698) PMC4455815 25948691

[52] Angelini DR, Kikuchi M, Jockusch EL. 2009 Genetic patterning in the adult capitate antenna of the beetle *Tribolium castaneum*. Dev. Biol. 327, 240–251. (doi:10.1016/j.ydbio.2008.10.047) 19059230

[53] Prpic NM, Janssen R, Wigand B, Klingler M, Damen WGM. 2003 Gene expression in spider appendages reveals reversal of exd/hth spatial specificity, altered leg gap gene dynamics, and suggests divergent distal morphogen signaling. Dev. Biol. 264, 119–140. (doi:10.1016/j.ydbio.2003.08.002) 14623236

[54] Panganiban G, Nagy L, Carroll SB. 1994 The role of the Distal-less gene in the development and evolution of insect limbs. Curr. Biol. 4, 671–675. (doi:10.1016/s0960-9822(00)00151–2) 7953552

[55] Panganiban G, Sebring A, Nagy L, Carroll S. 1995 The development of crustacean limbs and the evolution of arthropods. Science 270, 1363–1366. (doi:10.1126/science.270.5240.1363) 7481825

[56] Schaeper ND, Prpic NM, Wimmer EA. 2009 A conserved function of the zinc finger transcription factor Sp8/9 in allometric appendage growth in the milkweed bug *Oncopeltus fasciatus*. Dev. Genes Evol. 219, 427–435. (doi:10.1007/s00427-009-0301-0) 19760183 PMC2773111

[57] Angelini DR, Kaufman TC. 2005 Insect appendages and comparative ontogenetics. Dev. Biol. 286, 57–77. (doi:10.1016/j.ydbio.2005.07.006) 16112665

[58] Klementz B 2025 Data from: Developmental system drift in the patterning of the arthropod tarsus. Dryad Digital Repository. (doi:10.5061/dryad.95×69p8zs) PMC13085155 41667105

[59] Klementz B, Neu S, Laumer E, Hinne I, Setton E, Verma N 2025 Supplementary material from: Developmental system drift in the patterning of the arthropod tarsus. Figshare. (doi:10.6084/m9.figshare.c.8225229) PMC13085155 41667105

