# Supplementary material for: Developmental system drift in the patterning of the arthropod tarsus
Source: Proc Biol Sci. Author manuscript; Available in PMC 2026 Apr 16. (PMC13085155; doi:10.1098/rspb.2025.2557)
Supplement: Supplementary data [file NIHMS2154831-supplement-Supplementary_data.pdf]

Electronic Supplementary Material

Proceedings of the Royal Society B
DOI: 10.1098/rspb.2025.2557

Developmental system drift in the patterning of the arthropod tarsus

Benjamin C. Klementz[1]*, Sophie M. Neu[1], Ethan M. Laumer[1], Isaac A. Hinne[2], Emily V.W. Setton[3], Neeharika Verma[4], Max Hämmerle[5], John Rallis[6,7], Austen A. Barnett[8], Georg Brenneis[5], Monika Gulia-Nuss[2], Anastasios Pavlopoulos[6], Prashant P. Sharma[1]

[1]University of Wisconsin-Madison, Department of Integrative Biology, Madison 53706, Wisconsin, United States
[2]University of Nevada, Reno, Department of Biochemistry and Molecular Biology, Reno 89557, Nevada, United States
[3]Whitney Laboratory for Marine Bioscience, University of Florida, Saint Augustine 32080, Florida, United States
[4]Marine Biological Laboratory, University of Chicago, Woods Hole 02543, Massachusetts, United States
[5]Unit Integrative Zoologie, Department Evolutionsbiologie, Universität Wien, Vienna, Austria
[6]Institute of Molecular Biology and Biotechnology, Foundation for Research and Technology Hellas, 70013 Heraklion, Crete, Greece
[7]Department of Biology, University of Crete, 70013 Heraklion, Crete, Greece
[8]DeSales University, Department of Biology, Center Valley 18034, Pennsylvania, United States

*Correspondence: bklementz@wisc.edu

This file includes:

Supplementary Figures S1 to S10
Supplementary Tables S1 to S14

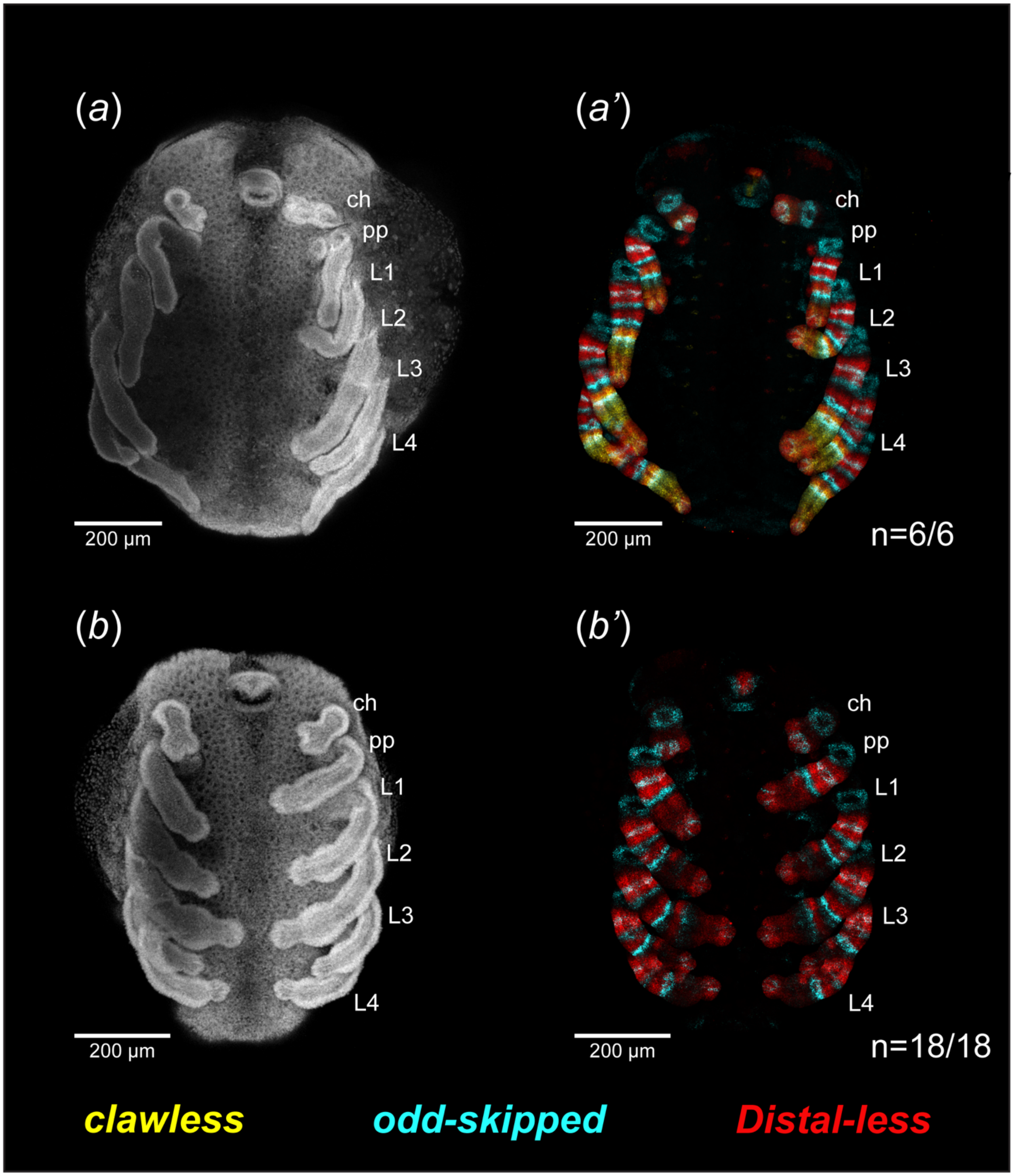

Figure S1. Gene expression assays reveal on-target depletions of *Po-cll* expression in RNAi treatments. (*a*) Stage 10 negative control *P. opilio* embryo with nuclear counterstaining (grey). (*a'*) Same embryo as in (a) with multiplexed expression of *Po-cll* (yellow), *Po-odd* (cyan), and *Po-Dll* (red). (*b*) Stage 10 *Po-cll* RNAi embryo with nuclear counterstaining. (*b'*) Same embryo as in (*b*) with multiplexed expression as in (*a'*). Note the absence of *Po-cll* expression. Abbreviations as in Figure 1.

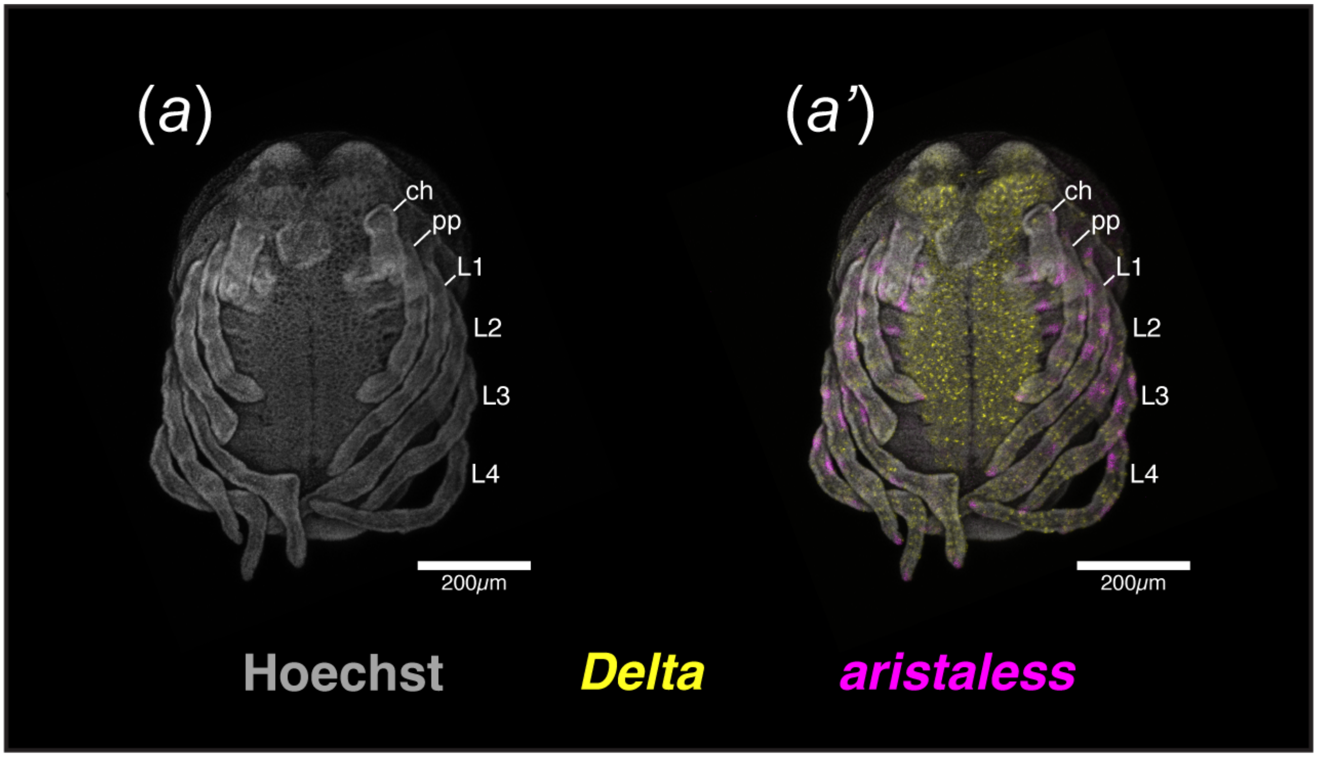

Figure S2. Severe *Po-cll* RNAi phenotypes are characterized by kinked appendages. (*a*) isolated nuclear counterstaining of stage 11 *Po-cll* RNAi embryo. (*a'*) Same embryo as in (a) with multiplexed expression of *Po-Dl* (yellow) and *Po-al* (magenta). Abbreviations as in Figure 1.

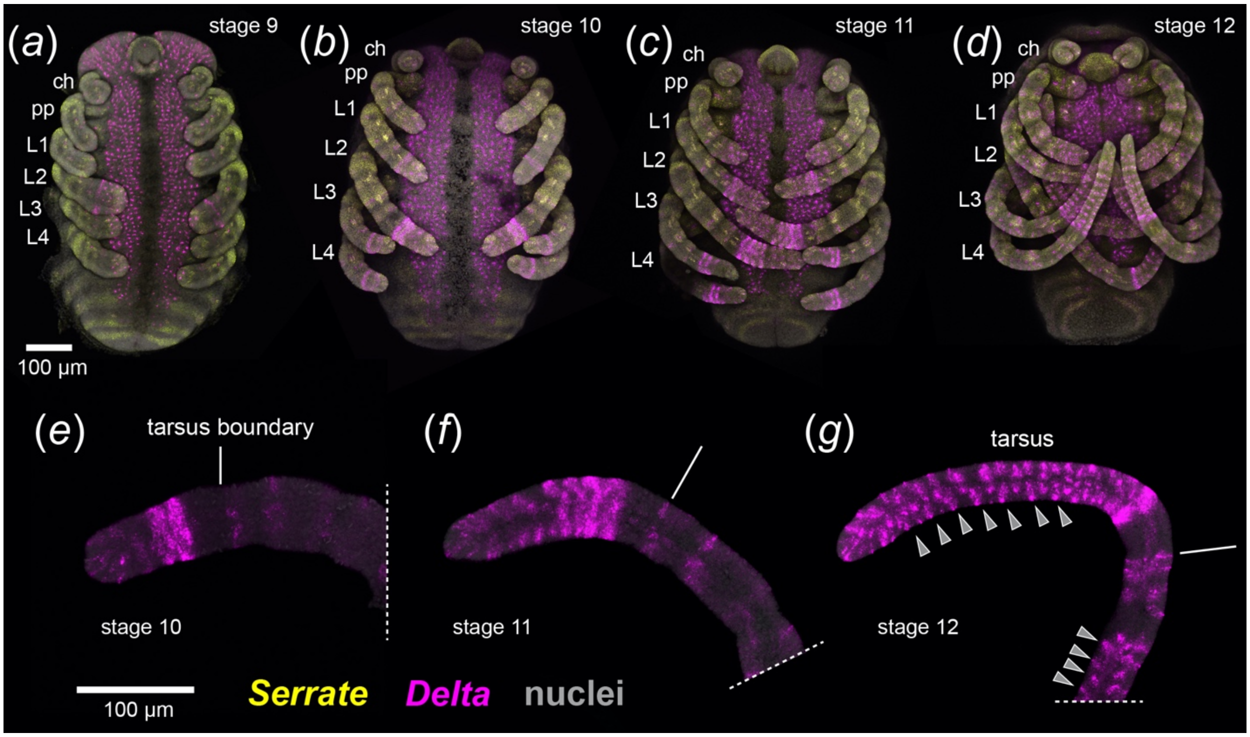

Figure S3. Characterization of *Po-Dl* and *Po-Ser* expression dynamics in the elongating tarsus of *P. opilio.* (*a-d*) Multiplexed expression of *Po-Dl* (magenta) and *Po-Ser* (yellow) with nuclear counterstaining (grey) in representative stages of tarsal elongation. (*a*) Stage 9 embryo. (*b*) Stage 10 embryo. (*c*) Stage 11 embryo. (*d*) Stage 12 embryo. (*e-g*) Leg II appendage mounts with isolated expression of *Po-Dl.* (*e*) Leg II of stage 10 embryo. (*f*) Leg II of stage 11 embryo. (*g*) Leg II of stage 12 embryo. Arrowheads: *Po-Dl* expression in putative sensory cells. Abbreviations as in Figure 1.

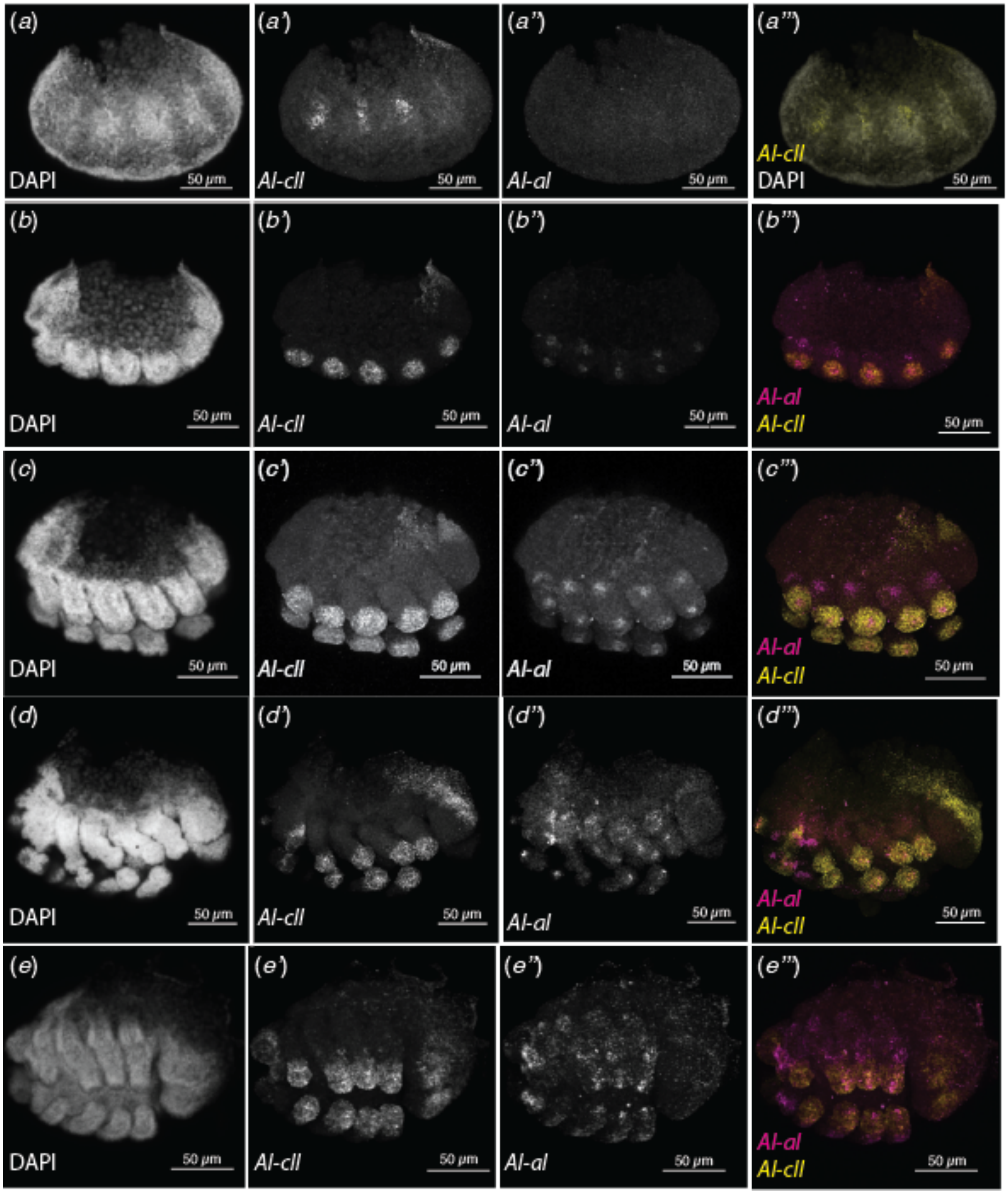

Figure S4. Expression dynamics of *Al-cll* and *Al-al* in representative stages of appendage formation and elongation in the acariform mite *A. longisetosus.* (*a-e*) Nuclear counterstaining of successive limb bud stages. (*a'-e'*) Isolated expression of *Al-cll.* (*a''-e''*) Isolated expression of *Al-al*. (*a'''-b'''*) Multiplexed expression of *Al-cll* (yellow) and *Al-al* (magenta).

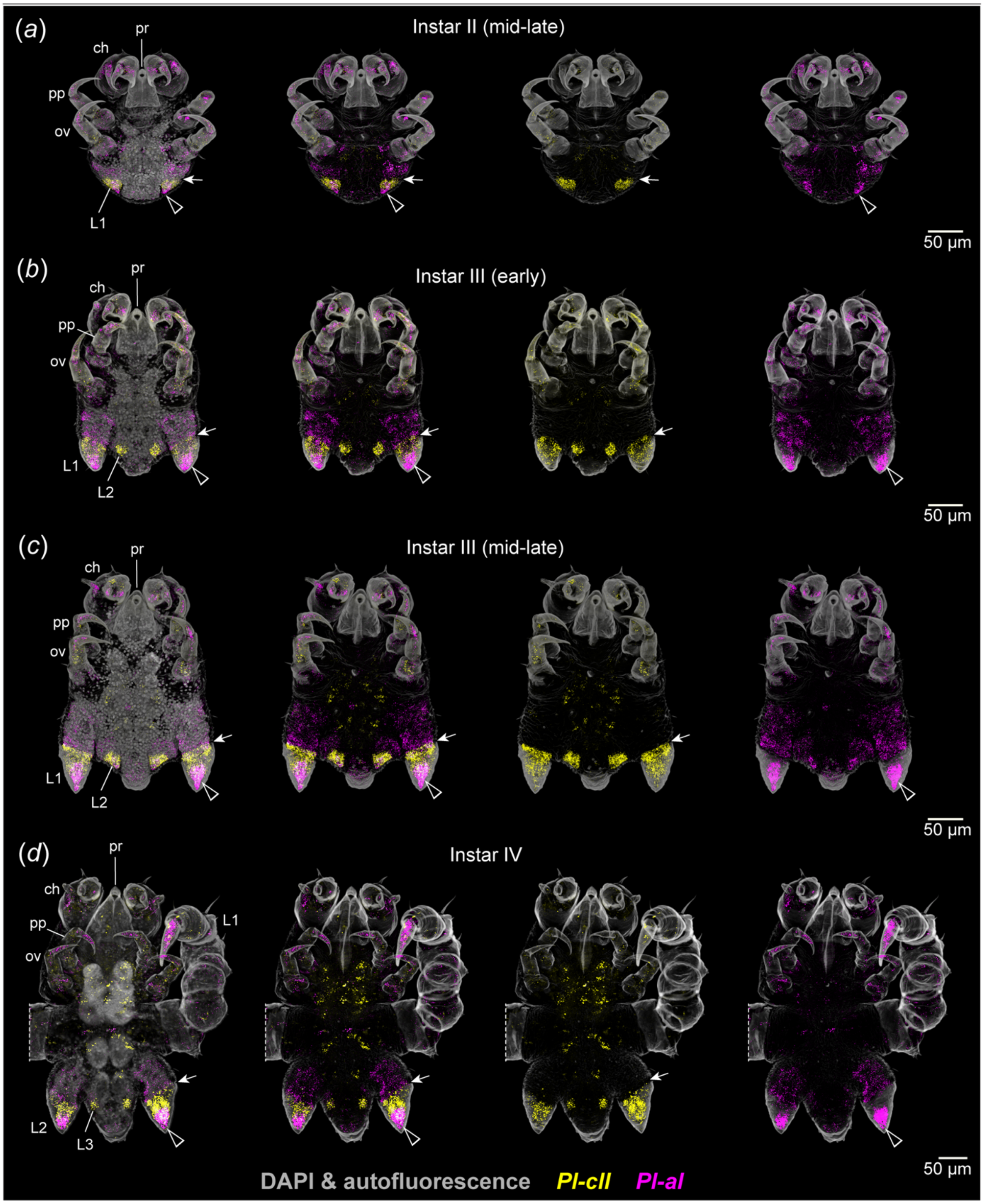

Figure S5. *Pl-cll* and *Pl-al* expression during postembryonic instars II-IV of the sea spider *P. litorale*. Maximum intensity projections of CLSM stacks in ventral view, cuticular autofluorescence and nuclear counterstaining in grey. (*a*) Instar II. (*b*) Early instar III. (*c*) Advanced instar III. (*d*) Instar IV. Abbreviations: ov – ovigeral larval limb; pr – proboscis; others as in Figure 1. Black arrowhead: *Pl-al* positive domain in the distal tip of limb buds. Arrow: sharp proximal boundary of *Pl-cll* expression in limb buds.

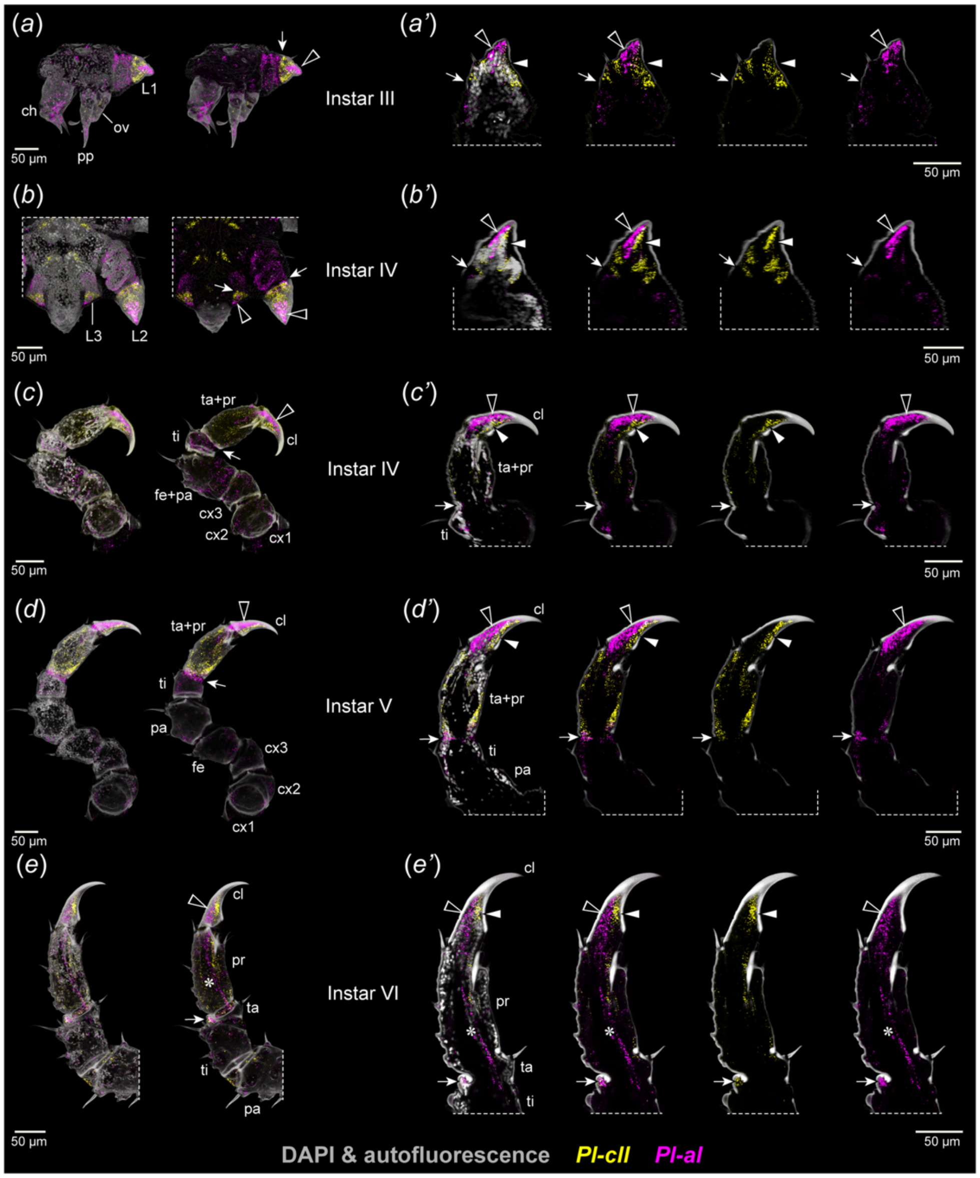

Figure S6. Details of *Pl-cll* and *Pl-al* expression dynamics during leg development of the sea spider *P. litorale.* Cuticular autofluorescence and nuclear counterstaining in grey. (*a-e*) Maximum intensity projections of complete CLSM stacks. (*a'-e'*) Extended virtual sections through limb buds and legs with dorsal side pointing left. (*a,a'*) Instar III in lateral view (*a*) and leg 1 bud (*a'*). (*b,b'*) Instar IV. Ventral view of posterior body pole (*b*) and pre-molting leg 2 bud with internally folded leg tissue (*b'*). (*c,c'*) Instar IV. Leg 1 (*c*) and detail of its distal podomeres (*c'*). (*d,d'*) Advanced instar V. Leg 1 (*d*) and detail of its distal podomeres (*d'*). (*e,e'*) Instar VI, distal podomeres of leg 1. Abbreviations: ov – ovigeral larval limb; others as in Figure 4. Black arrowhead: dorsal *Pl-al* domain in the distal limb bud tips and in the main claw of legs. White arrowhead: ventral *Pl-cll* domain in the distal limb bud tips and in the main claw of legs. Arrow: proximal boundary of *Pl-cll* expression in limb buds and region of *Pl-cll* and *Pl-al* co-expression at the tibial-tarsal boundary in developing legs. Asterisk: *Pl-al* positive (putatively mesodermal) cell strand in the propodus of instar VI.

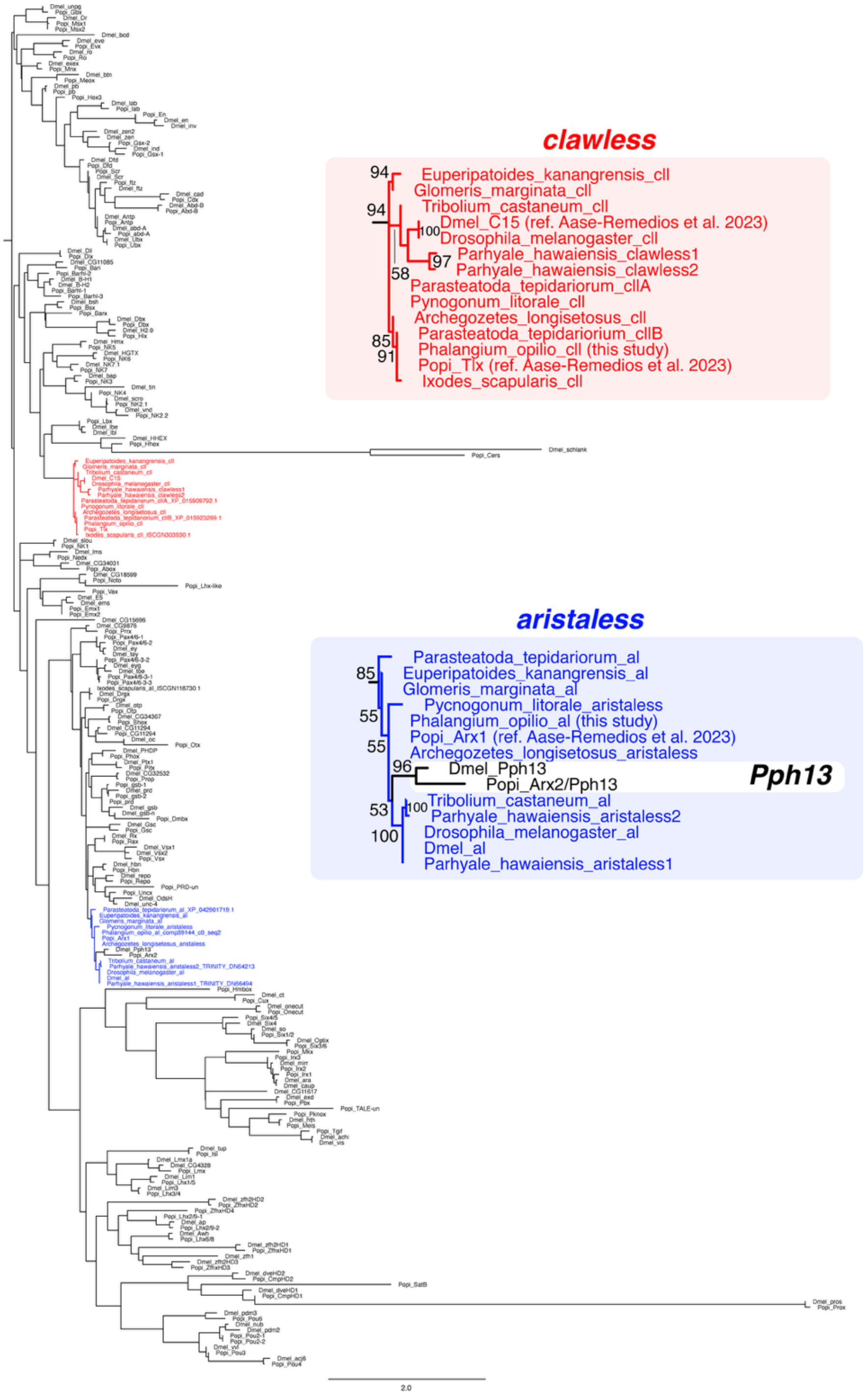

Figure S7. Maximum likelihood gene tree of the homeobox domain, sampling all homeobox genes of *D. melanogaster* and *P. opilio* (identified by Aase-Remedios et al. [23]) and supplemented with new identified *cll* and *al* sequences. Numbers on nodes correspond to ultrafast bootstrap resampling frequencies. Insets: Magnification of *cll* and *al* subtrees. Note the nested placement of *Pph13* in the *al* tree, as well as the complete sequence identity of *P. opilio cll* and *al* genes identified in this study and by Aase-Remedios et al. [23].

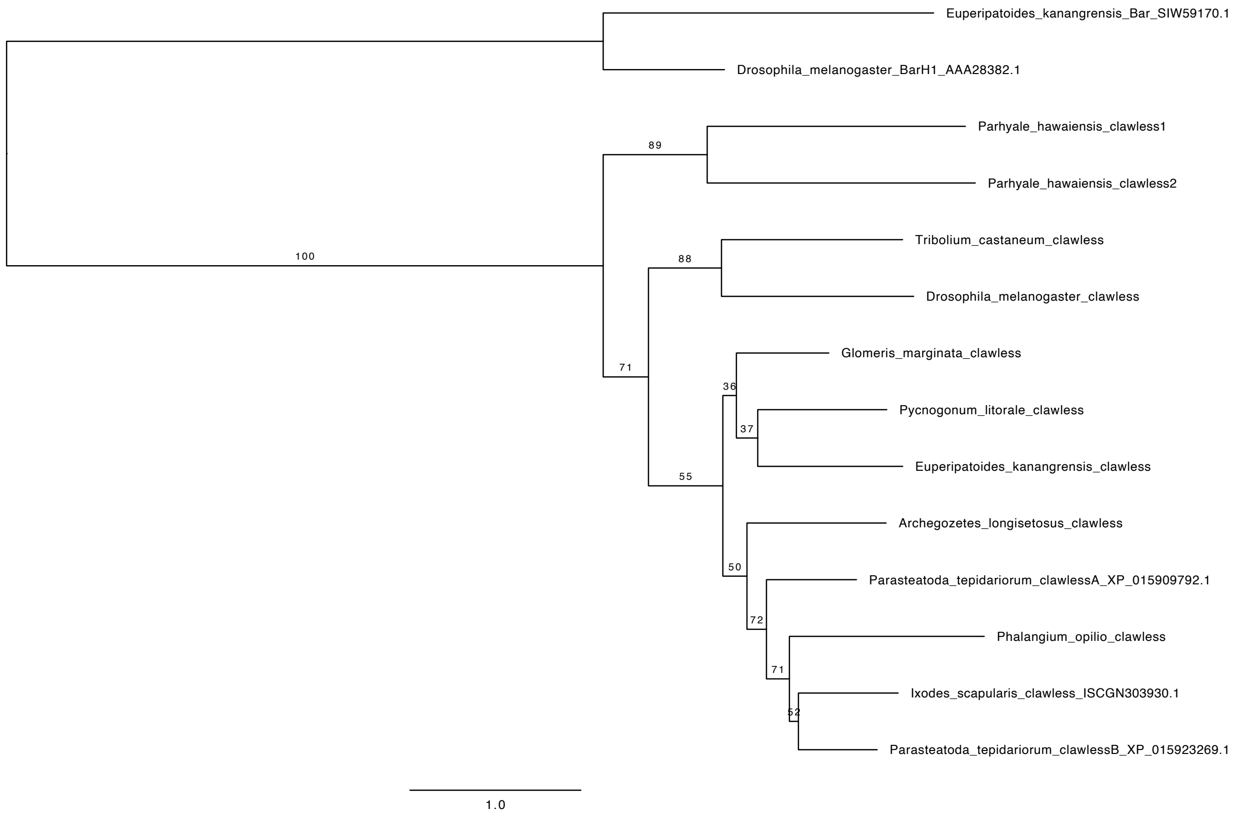

Figure S8. Maximum likelihood gene tree topology of arthropod *cll* homologs. Labels on branches represent ultrafast bootstrap support values.

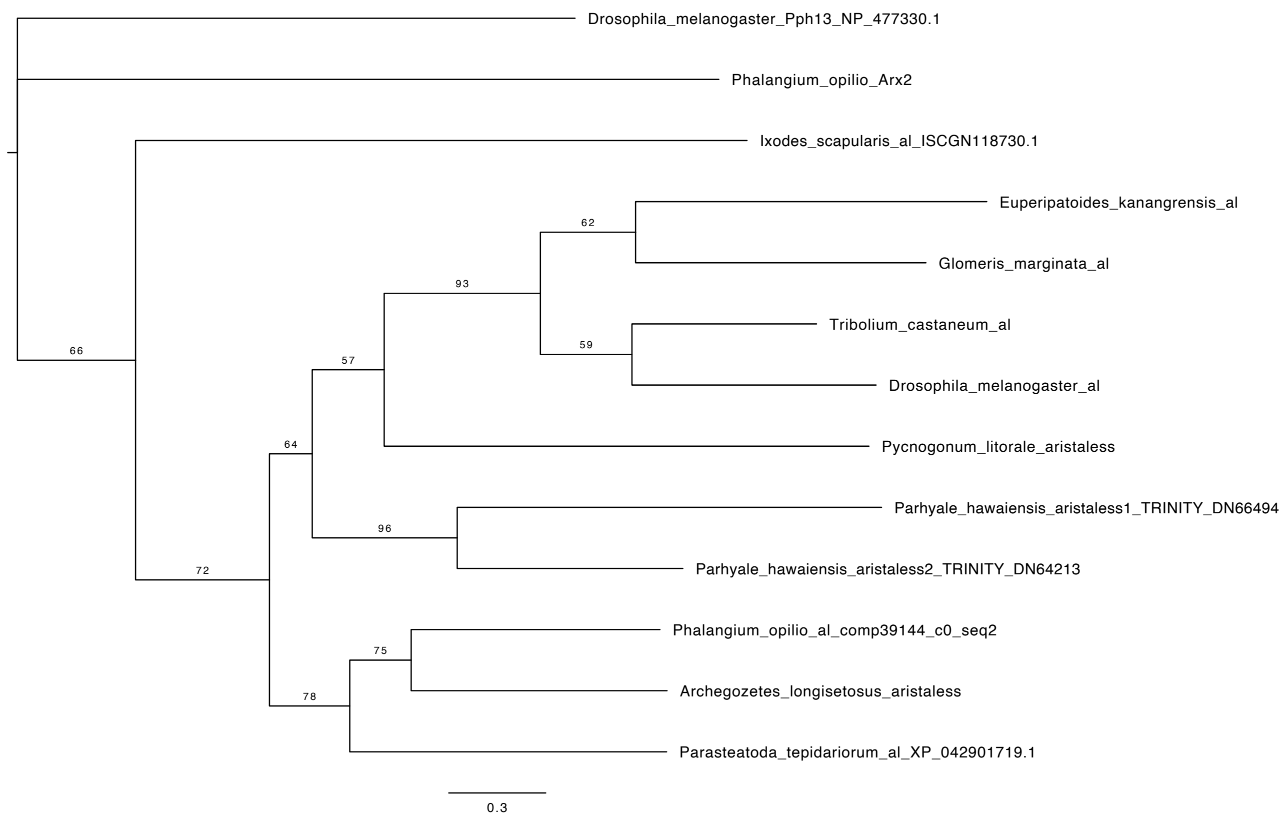

Figure S9. Maximum likelihood gene tree topology of arthropod *al* homologs. Labels on branches represent ultrafast bootstrap support values.

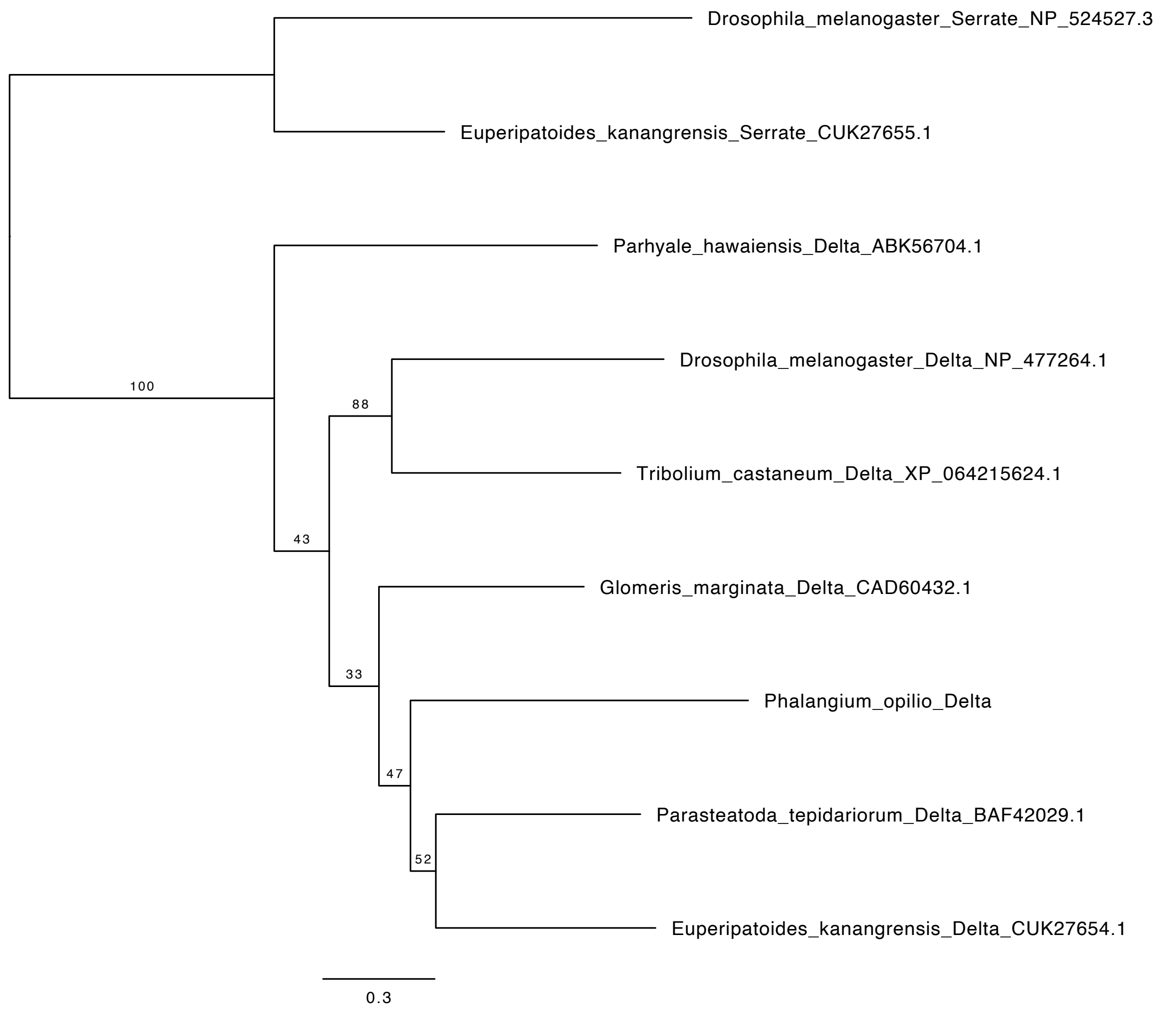

Figure S10. Maximum likelihood gene tree topology of arthropod *Dl* homologs. Labels on branches represent ultrafast bootstrap support values.

Table S1. Probe pairs designed for *Phalangium opilio clawless* HCR *in situ* hybridization (B2 initiator).

Pair	Initiator	Spacer	Hybridization	Hybridization	Spacer	Initiator
1	CCTCGTAAATCCTCATCA	AA	ATGTACAAAATCGCGATACAACGCC	AAAAACTTAAACTAAGCGCTGGAAG	AA	ATCATCCAGTAAACCGCC
2	CCTCGTAAATCCTCATCA	AA	CGCAAAATGTTCCATAATGTTCAGC	TCCAAGCTGAATTTTTGTTGGTCAT	AA	ATCATCCAGTAAACCGCC
3	CCTCGTAAATCCTCATCA	AA	GCGCGTGTTCGAATTGTTTTAAACG	ATTAAAACACATGAAATTCTGGTAG	AA	ATCATCCAGTAAACCGCC
4	CCTCGTAAATCCTCATCA	AA	GTCGGTATTTTTAGTGTGGGAAAAA	AAAAAGGCGTGTTTAGCGTGTGACT	AA	ATCATCCAGTAAACCGCC
5	CCTCGTAAATCCTCATCA	AA	TCGGTTCTTTAGTACAATCGGAGGC	ATAATGATTTTTGTCCGTTTTCCGT	AA	ATCATCCAGTAAACCGCC
6	CCTCGTAAATCCTCATCA	AA	AATCGGAGGAATGTTTTAGTTTTAG	GTCCGAAAAATCGCGAAGCTCAATT	AA	ATCATCCAGTAAACCGCC
7	CCTCGTAAATCCTCATCA	AA	GAAGCATTTGTTTAGCCAAATTATC	AGGTGGTCATAATTCGTACTGAAAC	AA	ATCATCCAGTAAACCGCC
8	CCTCGTAAATCCTCATCA	AA	TCTGGCTAAGAGAGCGCGCATTTTA	ACCTCAACAGTGCTTCGATGACCTT	AA	ATCATCCAGTAAACCGCC
9	CCTCGTAAATCCTCATCA	AA	AATTGGGTGTGGGTTTATGCGAGTG	TAATTATAGTGGTAATTATTCCTTC	AA	ATCATCCAGTAAACCGCC
10	CCTCGTAAATCCTCATCA	AA	CAAATAGGTGGTGATGTGATATACG	TTCGAATCACTAGTTTAGGTTATTC	AA	ATCATCCAGTAAACCGCC
11	CCTCGTAAATCCTCATCA	AA	ATGATGGTGATGATGATGGCCAAGA	TGGTCGATCGGCCGAAGAGTGCACG	AA	ATCATCCAGTAAACCGCC
12	CCTCGTAAATCCTCATCA	AA	ACCGTTTGGTGGTCCGCTTCCCGGT	TCCGTTTTGACCATCTTGACCCATT	AA	ATCATCCAGTAAACCGCC
13	CCTCGTAAATCCTCATCA	AA	GGAGGGACGCGTTACTCAGACACAA	CCCAAGGTTGTAGGTTTTGTAGCGC	AA	ATCATCCAGTAAACCGCC
14	CCTCGTAAATCCTCATCA	AA	CCACTTCCGCTTGCAAGGACATCAT	TGGCCGACTCGTACAGGCTCTTGGA	AA	ATCATCCAGTAAACCGCC
15	CCTCGTAAATCCTCATCA	AA	GGCGGTTTGCCTTCTCCATTTAGTT	CGCTTGCCTTTCCGCTTCGCGTTCT	AA	ATCATCCAGTAAACCGCC
16	CCTCGTAAATCCTCATCA	AA	GCATCGGTCATTTTGAGTTGCTTGG	CTGTTCTGAAACCACGTCTTGACCT	AA	ATCATCCAGTAAACCGCC
17	CCTCGTAAATCCTCATCA	AA	ACTTTTGTTTGTGAAACCTCTTCTC	GAGCGGCCCTTTCGGCGGACGCCAA	AA	ATCATCCAGTAAACCGCC
18	CCTCGTAAATCCTCATCA	AA	CGTCCTGGGCTTTTTCCGTTTGGGA	TTCGCATATTTGCATTCTCGTAAAG	AA	ATCATCCAGTAAACCGCC
19	CCTCGTAAATCCTCATCA	AA	GCAAAGAGAATGGACCTGGTAGTCT	TCACCGGTGGTAACGCCGCTGATGA	AA	ATCATCCAGTAAACCGCC
20	CCTCGTAAATCCTCATCA	AA	TTCAGCTGTTGCGCGTGATGCTGAT	GCGCCTAAGACGGAGAACTTGAGTT	AA	ATCATCCAGTAAACCGCC

Table S2. Probe pairs designed for *Phalangium opilio aristaless* HCR *in situ* hybridization (B1 initiator).

Pair	Initiator	Spacer	Hybridization	Hybridization	Spacer	Initiator
1	GAGGAGGGCAGCA AACGG	AA	TGGATTATATCCTTGATG GGCTTGG	TACTCCCGGTGGTACACCC GTTGAA	TA	GAAGAGTCTTCC TTTACG
2	GAGGAGGGCAGCA AACGG	AA	TTTGCACGACGATTTTGA AACCATA	CCAGCGGCCTTTTCTTGTTT ACGCC	TA	GAAGAGTCTTCC TTTACG
3	GAGGAGGGCAGCA AACGG	AA	TCATAGCCAGTTCTTCTCT TGTGAA	GAACTCGTGCTTCGGTTAA ATCGAC	TA	GAAGAGTCTTCC TTTACG
4	GAGGAGGGCAGCA AACGG	AA	TTTCTCAAGTTCTTCTAGT TGAAAA	GTCGGGATAGTGCGTCCTG CTGAAT	TA	GAAGAGTCTTCC TTTACG
5	GAGGAGGGCAGCA AACGG	AA	TTTCTTTTGGGAAAGTCCT CGGGTT	GTAAAGGTAGTTCTGTAAC GTCTTT	TA	GAAGAGTCTTCC TTTACG
6	GAGGAGGGCAGCA AACGG	AA	GGTTTGGACGTCCCGAAG AGTTAAC	TTCCTCCACCGCCGCCGCC ACCATT	TA	GAAGAGTCTTCC TTTACG
7	GAGGAGGGCAGCA AACGG	AA	CGGACTACCTGCATCTTG TTGTCTC	ATTGGCATCCCCGTTGTTG CTCAAC	TA	GAAGAGTCTTCC TTTACG
8	GAGGAGGGCAGCA AACGG	AA	TTAACCACGTCATCGCGT ATCCCGT	CAATCCATCGGAGATTCCG ACCGAT	TA	GAAGAGTCTTCC TTTACG
9	GAGGAGGGCAGCA AACGG	AA	TGTCCTCCGCGGAATTTCT TCGAGT	CAACCGAAAACTCTCCGTC GGAACA	TA	GAAGAGTCTTCC TTTACG
10	GAGGAGGGCAGCA AACGG	AA	GAATATTGTTAAGACCAT GAACGCC	CGTGATGACTGTTATTGTT ATTTTC	TA	GAAGAGTCTTCC TTTACG
11	GAGGAGGGCAGCA AACGG	AA	TAACGCGTTTGGACTTCC GGTTGTG	ACTTTTAAGTATTTTCTCCG CACTA	TA	GAAGAGTCTTCC TTTACG
12	GAGGAGGGCAGCA AACGG	AA	GCGCCAACGTGACGTAAG AATTGTT	GGACGCGGCGATGGTCCAC CACCCG	TA	GAAGAGTCTTCC TTTACG
13	GAGGAGGGCAGCA AACGG	AA	GGAATAGTTCGAAGTTAC GTTGATG	GCTGGTGCAAGTTTTGTAA TTGTTG	TA	GAAGAGTCTTCC TTTACG
14	GAGGAGGGCAGCA AACGG	AA	ATGATGGTAATTGTTCCC GAGCATT	GGCCGCCGCCGCTAAATGG TGCGGG	TA	GAAGAGTCTTCC TTTACG
15	GAGGAGGGCAGCA AACGG	AA	CGTGCGGATTCGTGGGGA TGATTAG	TCACTGGCTGATGTTAAGC CTAACG	TA	GAAGAGTCTTCC TTTACG
16	GAGGAGGGCAGCA AACGG	AA	TCCCCGTAACGGCAACGC GATTGTT	CCGATACACCCATGGTTGT TGTCGA	TA	GAAGAGTCTTCC TTTACG

Table S3. Probe pairs designed for *Phalangium opilio Delta* HCR *in situ* hybridization (B2 initiator).

Pair	Initiator	Spacer	Hybridzation	Hybridzation2	Spacer3	Initiator4
1	CCTCGTAAATCC TCATCA	AA	CGCGCACGTATAAGATCC TTGTCCC	ATTGGTTCCCGAAAATCC ATCAGGA	AA	ATCATCCAGTAA ACCGCC
2	CCTCGTAAATCC TCATCA	AA	GGCTTGTGATTTGTGCAA TAATTGA	TTCGTACAAGTACCGCCG TTCTTAC	AA	ATCATCCAGTAA ACCGCC
3	CCTCGTAAATCC TCATCA	AA	CTTCGTCGCACGTACACT GCCAAGG	CTTGGTTACAAAAGAGAC CACCCCA	AA	ATCATCCAGTAA ACCGCC
4	CCTCGTAAATCC TCATCA	AA	ATAGCGAATACATTCGTC GCAACGA	GTTGCAAGTTCCGTGTTG ACAACCA	AA	ATCATCCAGTAA ACCGCC
5	CCTCGTAAATCC TCATCA	AA	TCGTTTGGCTTATTGCAG GTACCGT	CCTTGCCAACCTGTCCTA CAAACGC	AA	ATCATCCAGTAA ACCGCC
6	CCTCGTAAATCC TCATCA	AA	CTTTGGTACAATATTCGC CACTCCA	GTTGGTGGCAACCAGGAG CGCATAT	AA	ATCATCCAGTAA ACCGCC
7	CCTCGTAAATCC TCATCA	AA	ACTACACGTATAATGTCC GAACTTG	AGGTAGACAGACTGTTTT ACCCTCG	AA	ATCATCCAGTAA ACCGCC
8	CCTCGTAAATCC TCATCA	AA	CCTTTACCGTAGTAATTA GGTTGGC	TCTCTGGGTCGACACAAC TTGGTGC	AA	ATCATCCAGTAA ACCGCC
9	CCTCGTAAATCC TCATCA	AA	CTTTACTGGTCTTGTTGT AATGTTC	GAACGCGGTAGGCGTACT TGACGGC	AA	ATCATCCAGTAA ACCGCC
10	CCTCGTAAATCC TCATCA	AA	AGGTGTCGCCAAACGAT AGATTAGA	CCAATTGGGACCGACGTC CAACCAA	AA	ATCATCCAGTAA ACCGCC
11	CCTCGTAAATCC TCATCA	AA	CCATTTCTGGAGTTCCAA GCTTCGA	GCATTGGGACCAGGTGAT GTAGAGT	AA	ATCATCCAGTAA ACCGCC
12	CCTCGTAAATCC TCATCA	AA	ATTCGAACTCGAATTTCA TGGGATT	TCAAGGAAAACGTACCCG TCCACGT	AA	ATCATCCAGTAA ACCGCC
13	CCTCGTAAATCC TCATCA	AA	GTTAAATGGAAAGATTAT GAGGCTA	GAACATTCCCGTCGAATC GGAGTTA	AA	ATCATCCAGTAA ACCGCC
14	CCTCGTAAATCC TCATCA	AA	ACGCCATTAGGATCGATG GCGACTT	GGGGTAACTTTGGAACCG AATGTGC	AA	ATCATCCAGTAA ACCGCC
15	CCTCGTAAATCC TCATCA	AA	TCCGGCAACTTCCGGTAC ACGATTT	AATGTTGAAGACATACTC GAAAGTA	AA	ATCATCCAGTAA ACCGCC
16	CCTCGTAAATCC TCATCA	AA	TCGAAATTCGACATTCTC AAAAGAA	GTCGCAACAATCTCCTTG CGCGTCT	AA	ATCATCCAGTAA ACCGCC
17	CCTCGTAAATCC TCATCA	AA	GAACATGACTGTGGCAAT AGAGTTA	AAACGTAGCTCAAAAACA CCTTCGC	AA	ATCATCCAGTAA ACCGCC
18	CCTCGTAAATCC TCATCA	AA	ACGACGTTCGAGATCCAA TGGCAAT	GCAACGTCGTTGTCAAAA TAATAAC	AA	ATCATCCAGTAA ACCGCC

Table S4. Probe pairs designed for *Phalangium opilio Serrate* HCR *in situ* hybridization (B1 initiator).

Pair	Initiator	Spacer	Hybridzation	Hybridzation2	Spacer3	Initiator4
1	GAGGAGGGCAGCAAACGG	AA	TGTTTCAACTTTAACCTCGATAACG	AGTACTCGTTTCGGGACTGATTCGA	TA	GAAGAGTCTTCCTTTACG
2	GAGGAGGGCAGCAAACGG	AA	CTGCTGATTAAATCGGCCATTCTCT	GACAAGGCGACACTATCAGATAGTT	TA	GAAGAGTCTTCCTTTACG
3	GAGGAGGGCAGCAAACGG	AA	GTATGGAAGGTTCGGCTGTGGAAGA	CGGCTTCGCTTAAGACTTGAAAAGT	TA	GAAGAGTCTTCCTTTACG
4	GAGGAGGGCAGCAAACGG	AA	CCACATTGAACAATAATTGGGAACG	ACAGTATCATCTTCGTTCATTTTCA	TA	GAAGAGTCTTCCTTTACG
5	GAGGAGGGCAGCAAACGG	AA	GTCTAAGTTGGACGCAGACTTCTTC	GAGTGAAGTTGTGAAGTATTGGGAA	TA	GAAGAGTCTTCCTTTACG
6	GAGGAGGGCAGCAAACGG	AA	AGCGCAGTTATTGCTGAGAGTGGCG	ACTCTTATCAAATAATAGAGTAATT	TA	GAAGAGTCTTCCTTTACG
7	GAGGAGGGCAGCAAACGG	AA	TATGGTGAGGTTAACATTGGGTCGT	TTAGGTTCGCATTCGTTGTTGAGTT	TA	GAAGAGTCTTCCTTTACG
8	GAGGAGGGCAGCAAACGG	AA	CTCTGGGTTTACAAGGTGGCGTGAA	GGTCGTGTCCAATACGAGTACAAAT	TA	GAAGAGTCTTCCTTTACG
9	GAGGAGGGCAGCAAACGG	AA	CGAACAGATATGATTAGGTGGACAC	ATCAAGGGCACTTTGACGGTGAATA	TA	GAAGAGTCTTCCTTTACG
10	GAGGAGGGCAGCAAACGG	AA	GGAACACAGTTCGGATGTCCACACC	TGTACTCCGTCAATTGGGTTCTGAT	TA	GAAGAGTCTTCCTTTACG
11	GAGGAGGGCAGCAAACGG	AA	GCATCGACAAGTGTTACACTCCCCT	CTGAGTGCATGTCACGTGTCCACGG	TA	GAAGAGTCTTCCTTTACG
12	GAGGAGGGCAGCAAACGG	AA	TTTCCATCCCAGATACAAGAACCGG	ACCCAAGTACTGCCATCAGGTTGAG	TA	GAAGAGTCTTCCTTTACG
13	GAGGAGGGCAGCAAACGG	AA	ATCGGCCTCCTTTACCAGGTGGACA	TTACAATTGGTACAACATCACGACA	TA	GAAGAGTCTTCCTTTACG
14	GAGGAGGGCAGCAAACGG	AA	GCAGGTACTGCCATCGGCGCAGGGA	ACAAGTGACGTTATTGATCTCATCT	TA	GAAGAGTCTTCCTTTACG
15	GAGGAGGGCAGCAAACGG	AA	CATTTGGGTCCAGTAAAACCAGGCG	CTATTACATTCGTTTTTATTGATCC	TA	GAAGAGTCTTCCTTTACG
16	GAGGAGGGCAGCAAACGG	AA	CAATACAACGACCACCGTTCATACA	ATTCGCACAAGTACCAATTAACTCC	TA	GAAGAGTCTTCCTTTACG
17	GAGGAGGGCAGCAAACGG	AA	TTGACACTTATGTCCATCATAACCT	ATTAGGTGAGCAGTCGTCAACGTTG	TA	GAAGAGTCTTCCTTTACG
18	GAGGAGGGCAGCAAACGG	AA	ACCGTGACGCATGTTGCGCCATTTT	CGACAGACGCAACTGTACGAATTAC	TA	GAAGAGTCTTCCTTTACG
19	GAGGAGGGCAGCAAACGG	AA	TAGAACTCTGACATGTCTTTCCTTG	AAGGATTTGATCTGCAGCTTGGATT	TA	GAAGAGTCTTCCTTTACG
20	GAGGAGGGCAGCAAACGG	AA	GTCACCGATATCGACGCAACTACCG	ACCTTCTTTGCAATGACACACAAAA	TA	GAAGAGTCTTCCTTTACG
21	GAGGAGGGCAGCAAACGG	AA	TGTGTCTTAAATTGCAAATTTTTCC	TTTGACACGTCGTGATATCGCAATG	TA	GAAGAGTCTTCCTTTACG
22	GAGGAGGGCAGCAAACGG	AA	ATCATTGATTAAATCGTTGCAAACA	CCATGGATGTTTGCACTCGCATGTA	TA	GAAGAGTCTTCCTTTACG
23	GAGGAGGGCAGCAAACGG	AA	TCGTTCTTATTTATATTGCAAACGG	TCATGTCGACATGGATTCGGATCGC	TA	GAAGAGTCTTCCTTTACG
24	GAGGAGGGCAGCAAACGG	AA	GGAATGTATTAACACCATCGACGCA	CTTCCCATCCATCAGGACAAACGCA	TA	GAAGAGTCTTCCTTTACG
25	GAGGAGGGCAGCAAACGG	AA	GCAATCATTTATATTCTCGTGACAG	CCCACCGTTTTGACAAGGATTCGAT	TA	GAAGAGTCTTCCTTTACG
26	GAGGAGGGCAGCAAACGG	AA	AAGTAAAACCACCATTTGGTTCGCT	TGCCGCTGTACCCTGGATCGCAGAC	TA	GAAGAGTCTTCCTTTACG
27	GAGGAGGGCAGCAAACGG	AA	AGTTGTTACCATGTGGTATCTTGAA	GCATTTTCCGTGGTTACCGCAAACT	TA	GAAGAGTCTTCCTTTACG
28	GAGGAGGGCAGCAAACGG	AA	TCAGTTTCTGAACAAGGTGGATTGT	CCTGAAGAGACTGGTACCATACAAC	TA	GAAGAGTCTTCCTTTACG

Table S5. Probe pairs designed for *Phalangium opilio odd-skipped* HCR *in situ* hybridization (B1 initiator).

Pair	Initiator	Spacer	Hybridization	Hybridization	Spacer	Initiator
1	GAGGAGGGCAGCAAACGG	AA	ATCAACCGTTATCTCCCCGTCACTT	TTAAGAAAGTACCGATTCCTCGCAC	TA	GAAGAGTCTTCCTTTACG
2	GAGGAGGGCAGCAAACGG	AA	TCAAGGTATGTCGACGTAGGTCGCA	CCATTTCCAAGTGTCTTAGAGAATG	TA	GAAGAGTCTTCCTTTACG
3	GAGGAGGGCAGCAAACGG	AA	CGTTGATTGAAACTTCGTCCGCAAG	GTTAAAAGGTGCGTCTTGAGGTTGG	TA	GAAGAGTCTTCCTTTACG
4	GAGGAGGGCAGCAAACGG	AA	CGTGCATTTGAAAGGCTTTTCTTTG	TGATTGGCAAAAACCCTTGCCACAT	TA	GAAGAGTCTTCCTTTACG
5	GAGGAGGGCAGCAAACGG	AA	TATGCGTCCGTTCATGAATCAACAA	TATCGCAAGTGTATGGCCTCTCGTC	TA	GAAGAGTCTTCCTTTACG
6	GAGGAGGGCAGCAAACGG	AA	TACCACCCCTTTCCAAATGCAACAA	CACCACCACCGTGCAAGAACGCGTG	TA	GAAGAGTCTTCCTTTACG
7	GAGGAGGGCAGCAAACGG	AA	GCTGCCGCAAAGGAGTGTCCTCCGT	TGTTTATTTCCTCTAAAAATGGCTG	TA	GAAGAGTCTTCCTTTACG
8	GAGGAGGGCAGCAAACGG	AA	ATCGTGAAAGTTAAGTTCATTTTGC	AATGTAATTACCATCGTACGGGTAA	TA	GAAGAGTCTTCCTTTACG
9	GAGGAGGGCAGCAAACGG	AA	CGTCATCTGATAACCTTCCTCTAAC	ATCCTGATTCGTCATCTTCAACATC	TA	GAAGAGTCTTCCTTTACG
10	GAGGAGGGCAGCAAACGG	AA	GCCAATCTTGCGAAATCAAACCGCT	GGATCCGAAATTGTAATGGCGGAAT	TA	GAAGAGTCTTCCTTTACG
11	GAGGAGGGCAGCAAACGG	AA	ATGACGGAGGTGATAACCCTCCACC	ACGAAGATGAAGATGAACAATCTTC	TA	GAAGAGTCTTCCTTTACG
12	GAGGAGGGCAGCAAACGG	AA	CGGTCACGATTTTGGTACTGGTCTT	GGGGAAATTACATTCCGATGATAGT	TA	GAAGAGTCTTCCTTTACG
13	GAGGAGGGCAGCAAACGG	AA	GCGCCGCTCTTCATTAAGGTAAAAT	TCGTCGGGATTGTCGAGGGGAGATG	TA	GAAGAGTCTTCCTTTACG
14	GAGGAGGGCAGCAAACGG	AA	CGAGATTCTTCTCGACTTCCTACTA	GAGCTTCTAGAGCCTGCGGCGAGTT	TA	GAAGAGTCTTCCTTTACG
15	GAGGAGGGCAGCAAACGG	AA	GGGGACATCCTCTTATCATTGGGAT	CCCCTCCGATATGTTTCGAGACAGC	TA	GAAGAGTCTTCCTTTACG
16	GAGGAGGGCAGCAAACGG	AA	GGTAGTGTAATTGTTCAGGTTGATT	CGCTCGAGGGCCCGTTAAGTCCCGC	TA	GAAGAGTCTTCCTTTACG
17	GAGGAGGGCAGCAAACGG	AA	CTCGTTCCATTAGGACCACCACCTT	ACGGTCCGCGGAACGTATTCCAATA	TA	GAAGAGTCTTCCTTTACG
18	GAGGAGGGCAGCAAACGG	AA	TTAGTAAAGGATTGGCAGCGGTGGT	CCATTGGTGACTGTCCAAGGTATGG	TA	GAAGAGTCTTCCTTTACG
19	GAGGAGGGCAGCAAACGG	AA	ATTCGTGGGTAACGGAGTTAGTAGG	ACTCGATTTCGTTTGAGGTGGTATG	TA	GAAGAGTCTTCCTTTACG
20	GAGGAGGGCAGCAAACGG	AA	ACGCCAGTTGGGGTTAAGGGTGGTG	CATGGTGATGTTGAATGAAGAGAAG	TA	GAAGAGTCTTCCTTTACG

Table S6. Probe pairs designed for *Phalangium opilio Distal-less* HCR *in situ* hybridization (B3 initiator).

Pair	Initiator	Spacer	Hybridization	Hybridization	Spacer	Initiator
1	GTCCCTGCCTCTATATCT	TT	TCGAAGAACTCCAGCGATTGATTAT	TTTTTCCGATAGGTTTTCGGGATGA	TT	CCACTCAACTTTAACCCG
2	GTCCCTGCCTCTATATCT	TT	CCCGTGTGATGGTTGCGCATTTGTC	CATATGCACGATGTTCACGTGTTCA	TT	CCACTCAACTTTAACCCG
3	GTCCCTGCCTCTATATCT	TT	TCTGGGAACGGTAATAGCTCGTTCC	CCCGGTTTGGTTTACTTCTTGTCGC	TT	CCACTCAACTTTAACCCG
4	GTCCCTGCCTCTATATCT	TT	ATTCGTGAGTGATTGGTTTTTGTTT	GAACTCGAAGTAACGCGAAGAGGGT	TT	CCACTCAACTTTAACCCG
5	GTCCCTGCCTCTATATCT	TT	TTCACGGGGTTATGTTAAAATTTGG	AATATAAACCACGAGACGTGACGTA	TT	CCACTCAACTTTAACCCG
6	GTCCCTGCCTCTATATCT	TT	CAAGAATACTGTGGCATGTAACCGT	GTTAACGAAGGGTCCGCTTGGTGAT	TT	CCACTCAACTTTAACCCG
7	GTCCCTGCCTCTATATCT	TT	GATTCATGTCCCAAGAACTAATAGG	TATGCATGTTCATAGCGGCTTTCGC	TT	CCACTCAACTTTAACCCG
8	GTCCCTGCCTCTATATCT	TT	CCCGATTGGGTCATCATGCCCGATT	GCGTTGGACATGCTATTTACGGGCG	TT	CCACTCAACTTTAACCCG
9	GTCCCTGCCTCTATATCT	TT	TCGTTGCCGGGTTTGGTTGCTGTAA	AATGTCCTTCCGACGGAGTTTGAGG	TT	CCACTCAACTTTAACCCG
10	GTCCCTGCCTCTATATCT	TT	CTTGTTGTTGAGCTTTCAACATTTT	GACCGCCCGGGCCCGAATTCGGAGG	TT	CCACTCAACTTTAACCCG
11	GTCCCTGCCTCTATATCT	TT	GATTTTCACCTGCGTTTGCGTTAGA	GTACTTGGAACGGCGATTTTGGAAC	TT	CCACTCAACTTTAACCCG
12	GTCCCTGCCTCTATATCT	TT	GGTAACGCCAAGTACTGCGTTCTTT	AGAGACGCGGCTAATTCCGCTCTTT	TT	CCACTCAACTTTAACCCG
13	GTCCCTGCCTCTATATCT	TT	GACTCGAGTATATCGTTCTGGGCTT	ACCTTCTGTTTAATTGCTGTAATTG	TT	CCACTCAACTTTAACCCG
14	GTCCCTGCCTCTATATCT	TT	GTCTCTTCTAAACCAGATTTGTCGT	TTTTTGCCCTTTCCATTGACTCTCA	TT	CCACTCAACTTTAACCCG
15	GTCCCTGCCTCTATATCT	TT	TGGCGAGGTAATGGCTAGGATGAGT	CACACGAACCGACGTTGGTCGGATA	TT	CCACTCAACTTTAACCCG
16	GTCCCTGCCTCTATATCT	TT	GGAATTGTTCATGGAAAAGGGGTAG	CGAATTATGCAAACCAGAGTTTAAC	TT	CCACTCAACTTTAACCCG
17	GTCCCTGCCTCTATATCT	TT	GAATTTCCATAAAGGCCGACTTGTT	CATTCAGTCCGGCCGCCGCCTGCTG	TT	CCACTCAACTTTAACCCG
18	GTCCCTGCCTCTATATCT	TT	GGTGGCGGCTTTGGCGGAACAACGT	AGCCGAGAACTCGCTAAAATTCAAC	TT	CCACTCAACTTTAACCCG

Table S7. Probe pairs designed for *Archegozetes longisetosus clawless* HCR *in situ* hybridization (B2 initiator).

Pair	Initiator	Spacer	Hybridization	Hybridization	Spacer	Initiator
1	CCTCGTAAATCCT CATCA	AA	GTAAGTTGTACTGTACAG AGATTTC	AAATGTTTGATAGCGATCG ATGCAA	AA	ATCATCCAGTAAA CCGCC
2	CCTCGTAAATCCT CATCA	AA	TGATGCCAACGGAAACGG TGGTCTA	ATTCGAGTCTAGTAACTAA CAGATT	AA	ATCATCCAGTAAA CCGCC
3	CCTCGTAAATCCT CATCA	AA	CCGTAGATCGATTTGGAT ACAACTT	GGGTGGTCAGATCCGGGC TGACCAG	AA	ATCATCCAGTAAA CCGCC
4	CCTCGTAAATCCT CATCA	AA	CTGCCTGACGTTCAGCCT CTCTTTC	CCTGTAATGACATCATCAG TCTATT	AA	ATCATCCAGTAAA CCGCC
5	CCTCGTAAATCCT CATCA	AA	CCTATTTTGAAACCAAGT CTTGACT	TGCTGTCTGACGTCTCCAC TTTGTT	AA	ATCATCCAGTAAA CCGCC
6	CCTCGTAAATCCT CATCA	AA	AGTGATGCTCTTTCAGCA GATGCTA	GCGTCTGTCATTTTCAATT GTTTGG	AA	ATCATCCAGTAAA CCGCC
7	CCTCGTAAATCCT CATCA	AA	TTGCATTCTTGTGAATGAT GTGCGC	GTGAAATCTCTTTTCTAGT TCACAA	AA	ATCATCCAGTAAA CCGCC
8	CCTCGTAAATCCT CATCA	AA	TGATAGGGATGACCTATT CTTCGAG	TTCTTCCTCTTTGGTGGTG TACGGT	AA	ATCATCCAGTAAA CCGCC
9	CCTCGTAAATCCT CATCA	AA	GAAAGGGCATTTGTAAAC CGTCTTT	GTACTGGCGGTAGACCAG CGCCAAG	AA	ATCATCCAGTAAA CCGCC
10	CCTCGTAAATCCT CATCA	AA	TGGGTAATGTGTAGTGTA AGGCATA	AAGCGTTGGCGGAGTTGG TCCTAAC	AA	ATCATCCAGTAAA CCGCC
11	CCTCGTAAATCCT CATCA	AA	CCGGTCAGATGATTCAAT GCTGCCT	ACACGTATGACCGAAGCG GCACCTG	AA	ATCATCCAGTAAA CCGCC
12	CCTCGTAAATCCT CATCA	AA	TCAGATTACATTTAGCAT CAGGTGA	ATGGACTCACTGTCACACG GCTTGT	AA	ATCATCCAGTAAA CCGCC
13	CCTCGTAAATCCT CATCA	AA	ATTGTTTTCAGTTGATGG ACATTGA	CGAAGAGGGGTATGAGGC AGCGGTC	AA	ATCATCCAGTAAA CCGCC
14	CCTCGTAAATCCT CATCA	AA	CCTTCTGAAGTAGCGCCG CTGTGAT	CAAAGCGATGACGGAGAC TCTGATT	AA	ATCATCCAGTAAA CCGCC
15	CCTCGTAAATCCT CATCA	AA	ATAGTCGACTTATACTGA ATGACAG	GTTTGTTTGAGCCACTATT TGCACC	AA	ATCATCCAGTAAA CCGCC
16	CCTCGTAAATCCT CATCA	AA	GTTTTGATGCACTGGTGA TGAGAGT	CGATGGTTTGCTAGAATTG ATTGAA	AA	ATCATCCAGTAAA CCGCC
17	CCTCGTAAATCCT CATCA	AA	TCCTCATCATCGTTAATGT CAATAC	TCTCGCTTTGGCGATCGAG ACATAT	AA	ATCATCCAGTAAA CCGCC
18	CCTCGTAAATCCT CATCA	AA	CTGATCGCGGCATTGAGT CCTCGTA	CATCTGAGTTTATGGGACT CGAAGA	AA	ATCATCCAGTAAA CCGCC
19	CCTCGTAAATCCT CATCA	AA	TGTTGTGATGTTCACTTAC AGGTAC	GATGAGAACAATTAAAAG CACATTT	AA	ATCATCCAGTAAA CCGCC
20	CCTCGTAAATCCT CATCA	AA	TTGTTGTCTGCGTAAAAG TTAACGC	TTTGAGAAACAACTCCAA AACAAAC	AA	ATCATCCAGTAAA CCGCC

Table S8. Probe pairs designed for *Archegozetes longisetosus aristaless* HCR *in situ* hybridization (B3 initiator).

Pair	Initiator	Spacer	Hybridization	Hybridization	Spacer	Initiator
1	GTCCCTGCCTCTATATCT	TT	ATTGATGGGATCACAGAAAAAGTGT	TTTTTGTTGCCATTAATCAATGGAC	TT	CCACTCAACTTTAACCCG
2	GTCCCTGCCTCTATATCT	TT	TCGTGTTCGCGTGCTTTCAGTCGGA	GGGTCTTTGCTTCGCATAACTGCCA	TT	CCACTCAACTTTAACCCG
3	GTCCCTGCCTCTATATCT	TT	AGTCGCAGAGGAGGTGTCTGTCTGT	AGACCTTCGTTTGGACTCGAGTTCG	TT	CCACTCAACTTTAACCCG
4	GTCCCTGCCTCTATATCT	TT	AGAGAGAGATGCGAGAAGACTTTGG	TCCTATTTTGGGCCTATTATGTGAT	TT	CCACTCAACTTTAACCCG
5	GTCCCTGCCTCTATATCT	TT	GTTGAGGATATGCTGCCGTCAGTTC	TGTTTGCGGACGGATTGCCGTGAAA	TT	CCACTCAACTTTAACCCG
6	GTCCCTGCCTCTATATCT	TT	TTAAGGCTTGGATGATCTAATAGTG	GCTGGAGTATTCAAGTAACCCGCTG	TT	CCACTCAACTTTAACCCG
7	GTCCCTGCCTCTATATCT	TT	GTTGCACCACTCGGTGTTCCGCCAC	TTGGTGATCACTTGTGATGGCAACA	TT	CCACTCAACTTTAACCCG
8	GTCCCTGCCTCTATATCT	TT	TCGCAAGTTCCTCCCTCGTAAATAC	CTCGTGCTTCAGTTAAATCGACTCG	TT	CCACTCAACTTTAACCCG
9	GTCCCTGCCTCTATATCT	TT	CGTTTGGGGAAATCATCGGAATGAT	AAAGTCGTTCGATATCTTCGCTGCT	TT	CCACTCAACTTTAACCCG
10	GTCCCTGCCTCTATATCT	TT	CTAACTCGTCAGGACCGCTACGGGG	CACCACTGTTTCTAATGGTCTGTGA	TT	CCACTCAACTTTAACCCG
11	GTCCCTGCCTCTATATCT	TT	ATCGCGCTCGTCATCATCTACTTTG	CAAAGGATTATCGTCATCATCCGAA	TT	CCACTCAACTTTAACCCG
12	GTCCCTGCCTCTATATCT	TT	GACCGGGGATACAACGCCCTTATTA	GTCGTCATCAACATCGGATCTGCTC	TT	CCACTCAACTTTAACCCG
13	GTCCCTGCCTCTATATCT	TT	CACATCTTTCAAGTACTCCCATTCC	TTTTGTCACTTAATAAGTTTTTCGG	TT	CCACTCAACTTTAACCCG
14	GTCCCTGCCTCTATATCT	TT	GTTGTTATTGAGCCAATTGTTGGCG	GATCTCTTAATAGCCTTATTATTAT	TT	CCACTCAACTTTAACCCG
15	GTCCCTGCCTCTATATCT	TT	AAGACAGCGCTTCCGATTGTGTTGG	TGCAGCTGTGGGTCGGTTTGTTGAT	TT	CCACTCAACTTTAACCCG
16	GTCCCTGCCTCTATATCT	TT	TCGCATGAAATCCAGGCCTGTTGTA	TGGTGATGGTGTGGCCAAAGTGGTG	TT	CCACTCAACTTTAACCCG
17	GTCCCTGCCTCTATATCT	TT	GGCTTCTGGTGGTATGTAGTCGTTG	GACGGCGAAATGGACGGAATTATAT	TT	CCACTCAACTTTAACCCG
18	GTCCCTGCCTCTATATCT	TT	TTGACTCCTTTTCCAAACCAATTAT	TCGTGATTTCTGTCAAAGGAGCCAG	TT	CCACTCAACTTTAACCCG
19	GTCCCTGCCTCTATATCT	TT	AAGTTTTGGCAATTGGGTCTGAACC	CAGTGAGAAGTTGTTTGTTTTAAAG	TT	CCACTCAACTTTAACCCG
20	GTCCCTGCCTCTATATCT	TT	GTGAGGCACTCACACAGAAAGTCAT	TTTTTAATAAGAGAAGAACAGTTTG	TT	CCACTCAACTTTAACCCG

Table S9. Probe pairs designed for *Ixodes scapularis clawless* HCR *in situ* hybridization (B1 initiator).

Pair	Initiator	Spacer	Hybridization	Hybridization	Spacer	Initiator
1	GAGGAGGGCAGCA AACGG	AA	TCCCTGGCGCCCTCGTAG ATGGACT	AGCGAGGCGTTGCTCATG CAGAGCG	TA	GAAGAGTCTTCC TTTACG
2	GAGGAGGGCAGCA AACGG	AA	TGAGTCTGTTGGCGGCGT GGCGTTC	TCACCACCTCAGCCTGGA GTGACAT	TA	GAAGAGTCTTCC TTTACG
3	GAGGAGGGCAGCA AACGG	AA	ATCGTCAGGAAATGCCGA GTACGTT	TTCGCGCTCTTCGGCCGTC TGTCTC	TA	GAAGAGTCTTCC TTTACG
4	GAGGAGGGCAGCA AACGG	AA	CTGCGGTTCTGGAACCAC GTCTTGA	AGAGGGCTGATTTTTCTC CACTTTG	TA	GAAGAGTCTTCC TTTACG
5	GAGGAGGGCAGCA AACGG	AA	TGCTTGTGGAAGCGCTTCT CGAGCT	GCCCGCTCCGCACTGGCC AGGTACT	TA	GAAGAGTCTTCC TTTACG
6	GAGGAGGGCAGCA AACGG	AA	AAGGTGGAGCGGGCTGAG CTGTGCT	CACGCGAATCACAGTCCC GTTGGCA	TA	GAAGAGTCTTCC TTTACG

Table S10. Probe pairs designed for *Pycnogonum litorale clawless* HCR *in situ* hybridization (B5 initiator).

Pair	Initiator	Spacer	Hybridization	Hybridization	Spacer	Initiator
1	CTCACTCCCAAT CTCTAT	AA	CGATTCACCTGTAATGCTA ACCGTC	GGAATCATACGCTCGATG ATGCTAT	AA	CTACCCTACAAA TCCAAT
2	CTCACTCCCAAT CTCTAT	AA	AGGTAAATTACTGGCCTCA GTTAAA	TGCGGTGAGCACCACTGG AACGATT	AA	CTACCCTACAAA TCCAAT
3	CTCACTCCCAAT CTCTAT	AA	TATTGGAGAGGCAAGCGT TGGTACA	CTAAGATAAATCTGAAGT CGATCAG	AA	CTACCCTACAAA TCCAAT
4	CTCACTCCCAAT CTCTAT	AA	GCGTTGTCGTGATTATTTT CTTGAC	CTCAGATAAGACGATGTG GAATCTC	AA	CTACCCTACAAA TCCAAT
5	CTCACTCCCAAT CTCTAT	AA	TGTTAATACATAATGGATC ACCTCG	TGCTTTGCAAAGCGTGTAA TGAAGC	AA	CTACCCTACAAA TCCAAT
6	CTCACTCCCAAT CTCTAT	AA	TACAGCTTCTGCTTGTAAT GACATC	TGCAGGATCGCCATAGATT CCCTTG	AA	CTACCCTACAAA TCCAAT
7	CTCACTCCCAAT CTCTAT	AA	TCACGCTCTTCTGCTGTCT GTCTTC	AAACGATTAGCCGCTTGA CGCTCTG	AA	CTACCCTACAAA TCCAAT
8	CTCACTCCCAAT CTCTAT	AA	CTTTACTTGCGCATCGGTC ATCTTC	TTTAGTTCTCCTGTTTTGA AACCAC	AA	CTACCCTACAAA TCCAAT
9	CTCACTCCCAAT CTCTAT	AA	TTATGGAATCTCTTTTCCA ATTCGC	CTTTCAGCGGACGCTAAAT ATTTCT	AA	CTACCCTACAAA TCCAAT
10	CTCACTCCCAAT CTCTAT	AA	GCTTCTTACGTTTCGGAGG TGTTCT	TCTGCATCCGAGTGAAAG ATGTCCT	AA	CTACCCTACAAA TCCAAT
11	CTCACTCCCAAT CTCTAT	AA	CATCGGTACCGGAACTTGC ATTCTG	CTGATACGGGTGACCTATT CTTCTA	AA	CTACCCTACAAA TCCAAT
12	CTCACTCCCAAT CTCTAT	AA	GCCATCCAAGGAAACGCA GCTGAAG	TTCATTGTGAGAGCTAAAC TTCTTG	AA	CTACCCTACAAA TCCAAT
13	CTCACTCCCAAT CTCTAT	AA	TGTGCCGGTACCCTGATAA CAGTTC	GAAAACTGCGGCATTCCG ATCGGGT	AA	CTACCCTACAAA TCCAAT
14	CTCACTCCCAAT CTCTAT	AA	GTGATAAAGAATGCGACG TTATACC	GGGCACCACCAAAAAGTG GATCGTA	AA	CTACCCTACAAA TCCAAT
15	CTCACTCCCAAT CTCTAT	AA	ATCGGCGGTGATGCCATC GAACTGT	AGACTAGTAACCGGTATG TAAGCTC	AA	CTACCCTACAAA TCCAAT
16	CTCACTCCCAAT CTCTAT	AA	TACCGTTGCTACTTGCCGG TGACGA	TAAACGCCATGAAATGTTC AATCTG	AA	CTACCCTACAAA TCCAAT
17	CTCACTCCCAAT CTCTAT	AA	TTCGCCCAGTAGACGACTG ATACTG	TGCAGAGTTGTCATCTTTA TCTTCT	AA	CTACCCTACAAA TCCAAT
18	CTCACTCCCAAT CTCTAT	AA	GATGAAGATCTGTTTATTG TTGGAG	TTATTATTTGATGACGGTG AACGAG	AA	CTACCCTACAAA TCCAAT
19	CTCACTCCCAAT CTCTAT	AA	TGATTTCCATATTATCCGG ATCCAT	GGGGAGTTTTCTCCGACCC AACATC	AA	CTACCCTACAAA TCCAAT

Table S11. Probe pairs designed for *Pycnogonum litorale aristaless* HCR *in situ* hybridization (B2 initiator).

Pair	Initiator	Spacer	Hybridization	Hybridization	Spacer	Initiator
1	CCTCGTAAATCCTCATCA	AA	ATGTTACTGATTGTCTCATTGGCTC	TTTTTCAAAGTACGGTTCTCACTGG	AA	ATCATCCAGTAAACCGCC
2	CCTCGTAAATCCTCATCA	AA	TCCGCCCATACCTGACGTAGCGATA	ACTTCTGAGTTTTTCGGCATCCGCG	AA	ATCATCCAGTAAACCGCC
3	CCTCGTAAATCCTCATCA	AA	TCGCCATCGCTTGATGACGACTCTG	TGCTCGCACTTCCGGTATTCGCTGA	AA	ATCATCCAGTAAACCGCC
4	CCTCGTAAATCCTCATCA	AA	CGCCAACATTTCGGCCAACAGAATC	TTCGAGTTGTTGCATCGCCTCGCGT	AA	ATCATCCAGTAAACCGCC
5	CCTCGTAAATCCTCATCA	AA	CAACGAGGCACTCGGTCCAGGAAAT	CATCGAAGCGGCCGAATTGATGTGC	AA	ATCATCCAGTAAACCGCC
6	CCTCGTAAATCCTCATCA	AA	GTAGACCGGTCCATTGACGGAATCT	ATAACGAACCGGCCGTCATGGTGGA	AA	ATCATCCAGTAAACCGCC
7	CCTCGTAAATCCTCATCA	AA	AGTAAAGGCCATCGAGGCGGATCGT	CAGTTCTCATGAATGGATTGTACAT	AA	ATCATCCAGTAAACCGCC
8	CCTCGTAAATCCTCATCA	AA	TCGGTGGTACAGACTTACATTCTCG	AACTTGTGGAACGTGAGGAACTCTC	AA	ATCATCCAGTAAACCGCC
9	CCTCGTAAATCCTCATCA	AA	TTGATGCGCCGGAATCGGTCCTTGA	CTTGTCTAAAGACGGAAGCCATTTC	AA	ATCATCCAGTAAACCGCC
10	CCTCGTAAATCCTCATCA	AA	GGATGTTCGTGCGGACCGACTTTCT	GACGCGTTCGATGGAAATCCGGAAT	AA	ATCATCCAGTAAACCGCC
11	CCTCGTAAATCCTCATCA	AA	GGAACCAAACCTGCACTCTAGCTTC	GCTTTCGCCATTTGGCTCTCCTATT	AA	ATCATCCAGTAAACCGCC
12	CCTCGTAAATCCTCATCA	AA	TCGAGTAAACACATCCGGATAATGA	TAGATTAACTCTCATCGCCAATTCC	AA	ATCATCCAGTAAACCGCC
13	CCTCGTAAATCCTCATCA	AA	AGTTGAAAGCTGCTGAATGTGGTCC	CTTCCGAAGACCTTCTCCAGTTCTT	AA	ATCATCCAGTAAACCGCC
14	CCTCGTAAATCCTCATCA	AA	AATCGTCGAGATGATCGGGTGACGG	ACCTCCTCTGCTTCCTTTTTGGCGG	AA	ATCATCCAGTAAACCGCC
15	CCTCGTAAATCCTCATCA	AA	CGCGATTGACGACATCTTATGTTGG	GCCGTCCGATAATCGGCGATGCATT	AA	ATCATCCAGTAAACCGCC
16	CCTCGTAAATCCTCATCA	AA	ACGTCAACATCGTCGGTTACGGGTT	GATCGGTCGGTTTCGTCGCAGGTTA	AA	ATCATCCAGTAAACCGCC
17	CCTCGTAAATCCTCATCA	AA	CGGCTCCATAAAATATGATATTCCC	ACGGCGGGCGGATGGAGTTAGATTA	AA	ATCATCCAGTAAACCGCC
18	CCTCGTAAATCCTCATCA	AA	AGACAATGAGATCTGTAACAGTTAC	TAATTAATCTTGATGACGAGTTCCC	AA	ATCATCCAGTAAACCGCC
19	CCTCGTAAATCCTCATCA	AA	AACTCGTCTTCACGTGACGGTTAAC	GTTGTTGTATCGGATAATCGCGCGA	AA	ATCATCCAGTAAACCGCC

Table S12. Probe pairs designed for *Parhyale hawaiensis clawless-1* HCR *in situ* hybridization (B2 initiator).

Pair	Initiator	Spacer	Hybridization	Hybridization	Spacer	Initiator
1	CCTCGTAAATCCTCATCA	AA	AAACCAGAAGAAAGCTGAGATTGTG	GGGGACCTCAGGTTTTCCGATTTGT	AA	ATCATCCAGTAAACCGCC
2	CCTCGTAAATCCTCATCA	AA	GTTGTAACTGCTGATGAATACGAGA	GATGTTGCAACTGCTCCAGCTGCTG	AA	ATCATCCAGTAAACCGCC
3	CCTCGTAAATCCTCATCA	AA	ACGGAGGAGAGGGAGTGTGAAGAAT	AGTACCTCGAGTGGGGAATTGGGCC	AA	ATCATCCAGTAAACCGCC
4	CCTCGTAAATCCTCATCA	AA	GTTTTGTGCCTGTCTGTCTGTTTCA	GCCGGACGGCATGCCGATCATAAGA	AA	ATCATCCAGTAAACCGCC
5	CCTCGTAAATCCTCATCA	AA	GTCCTCCGGTTCTGGAACCACGTCT	TCTTCTGCTTCTTGGCGTCTCCACT	AA	ATCATCCAGTAAACCGCC
6	CCTCGTAAATCCTCATCA	AA	AGCACGTTCGCTGCTGGCCAGATAT	GGTCATCTTAAGCTGCTTGGCCAGT	AA	ATCATCCAGTAAACCGCC
7	CCTCGTAAATCCTCATCA	AA	TTGACCTGTACCCTCGTAAAAGAAG	TGTTTATTGAAGCGCTTCTCTAGTT	AA	ATCATCCAGTAAACCGCC
8	CCTCGTAAATCCTCATCA	AA	GGGAGGTACCAGGGAGTTGCCTAGA	CGCTAACCAAGGCAGTGTTGAGTTC	AA	ATCATCCAGTAAACCGCC
9	CCTCGTAAATCCTCATCA	AA	CTCATGAGGCTATTGGGATTGCCAA	GGAACTCTTATAACTCCGGCTGGGC	AA	ATCATCCAGTAAACCGCC
10	CCTCGTAAATCCTCATCA	AA	ACAATCCGTGTAAATGAGGTAACAA	CATGAGCTCCTAGGCCCAGAGACGG	AA	ATCATCCAGTAAACCGCC
11	CCTCGTAAATCCTCATCA	AA	GTAAGGTTGGAAGGGTCTATTGGGA	AGCAGCGAAGGGCATATGAGTATAT	AA	ATCATCCAGTAAACCGCC
12	CCTCGTAAATCCTCATCA	AA	TGCCGGTCGTCATGTCGGTCATCAT	TCTTCGTCCCCATTGCTCTCATCAT	AA	ATCATCCAGTAAACCGCC
13	CCTCGTAAATCCTCATCA	AA	TCCTTCACTTTCGCTGTGTTCTTGT	GATGCCATCGTCCTCTCCGTGAGCC	AA	ATCATCCAGTAAACCGCC
14	CCTCGTAAATCCTCATCA	AA	TCCTTTCCAGGGATTTTGTTCCTGT	AGGTCTCTTTCGCCACGGATTTCCA	AA	ATCATCCAGTAAACCGCC
15	CCTCGTAAATCCTCATCA	AA	CCCTGTCTCGCTCAAATAACTCCCG	GCAAATCTGTTTCTCTGTCCAGCAG	AA	ATCATCCAGTAAACCGCC
16	CCTCGTAAATCCTCATCA	AA	AAGGAAGTCGATAACAGCGAAGATA	TCGCGCGACGCGTTCAGTTGCGACG	AA	ATCATCCAGTAAACCGCC
17	CCTCGTAAATCCTCATCA	AA	CTCTCCTTAAATCGAGAGGTGAGGG	TGAAGGGCAGTCGAGGAGTGTGATC	AA	ATCATCCAGTAAACCGCC
18	CCTCGTAAATCCTCATCA	AA	TATTAGGGAAGGCTCGTCGCAAACT	AGGAGAGGAAGAGGTGGGTGGGAGA	AA	ATCATCCAGTAAACCGCC

Table S13. Probe pairs designed for *Parhyale hawaiensis clawless-2* HCR *in situ* hybridization (B2 initiator).

Pair	Initiator	Spacer	Hybridzation	Hybridzation2	Spacer3	Initiator4
1	CCTCGTAAATCCTCATCA	AA	AGGACTGGGACTTCGACTTCGAGAT	TTACGATGACCCCGGCGACATCATA	AA	ATCATCCAGTAAACCGCC
2	CCTCGTAAATCCTCATCA	AA	TTAGAAGGGAACCTTTCGTCGGCCT	CGACTTTGTATATCAAGACGATCGG	AA	ATCATCCAGTAAACCGCC
3	CCTCGTAAATCCTCATCA	AA	TCCTCTCAGCCCAAGGTTTGATATT	CACCACCAGAGGCCGCTACAGGTTT	AA	ATCATCCAGTAAACCGCC
4	CCTCGTAAATCCTCATCA	AA	AGGGCGTCTCATTGAGCAGACCGTT	ACGCGTTCGTATTGTAGAGCGGTGT	AA	ATCATCCAGTAAACCGCC
5	CCTCGTAAATCCTCATCA	AA	GTGCTGCCGCTCAGCCTCCCGTTCT	GTGTAGATTTAATAATATACGATTG	AA	ATCATCCAGTAAACCGCC
6	CCTCGTAAATCCTCATCA	AA	CTGTTTTGAAACCAAGTCTTGACCT	GCGATCTGGCGTCTCCATTTAGTTC	AA	ATCATCCAGTAAACCGCC
7	CCTCGTAAATCCTCATCA	AA	GAGGCTCGTTCGCTGCTGGCCAGAT	TCAGTCATACTTAGTGTTTTGGCCA	AA	ATCATCCAGTAAACCGCC
8	CCTCGTAAATCCTCATCA	AA	CATTGACCTGTACCCTGGTGAAGGA	TCTGTTTGTTGAAGCGCTTCTCCAG	AA	ATCATCCAGTAAACCGCC
9	CCTCGTAAATCCTCATCA	AA	AGTGTGTGGACTGAAGCTAAGGTGA	AGGTCTATGGGCTGGTACCCGAAGG	AA	ATCATCCAGTAAACCGCC
10	CCTCGTAAATCCTCATCA	AA	CTTGTTAACGTTGGGCCTAAATGAG	CCTGACGCTTGTATAGAACTGGCCA	AA	ATCATCCAGTAAACCGCC
11	CCTCGTAAATCCTCATCA	AA	TGATGCCCTCGTAATATTCTTCATA	AATAATTTGGCGGTAACGTTCCCCG	AA	ATCATCCAGTAAACCGCC
12	CCTCGTAAATCCTCATCA	AA	TCTCTATCGTCACCGTCGTGTCCAT	TCATCTTCATCTGCCCCGGCATTGC	AA	ATCATCCAGTAAACCGCC
13	CCTCGTAAATCCTCATCA	AA	TCCTCCTCTCTGTAGTTCCTGTGAT	TGCTCATCGTCAGAGAGCACCGACG	AA	ATCATCCAGTAAACCGCC
14	CCTCGTAAATCCTCATCA	AA	GCTTAATTGCTTTCGGCCGAAAGCT	CTTTATTGTATTCGTCCCCAGGCTT	AA	ATCATCCAGTAAACCGCC
15	CCTCGTAAATCCTCATCA	AA	CGTTGATGTCTTCCTCATGTTCCAA	GACTGCCGGGTCGGCTGTCAGAATC	AA	ATCATCCAGTAAACCGCC

Table S14. Primer pairs for designed for *Phalangium opilio clawless* RNAi experiments.

Primer name	Primer sequence	Amplicon length
Popi_cll_for	GCAACAGCTGAACGAACTCA	812 bp
Popi_cll_rev	TATACGTGGGTGGTCGATCG	
